# Anode Surface Bioaugmentation Enhances Deterministic Biofilm Assembly in Microbial Fuel Cells

**DOI:** 10.1128/mBio.03629-20

**Published:** 2021-03-02

**Authors:** Keren Yanuka-Golub, Vadim Dubinsky, Elisa Korenblum, Leah Reshef, Maya Ofek-Lalzar, Judith Rishpon, Uri Gophna

**Affiliations:** a The Porter School of Environmental Studies, Tel Aviv University, Tel Aviv, Israel; b The Shmunis School of Biomedicine and Cancer Research, The George S. Wise Faculty of Life Sciences, Tel Aviv University, Tel Aviv, Israel; c Department of Plant and Environmental Sciences, Weizmann Institute of Science, Rehovot, Israel; d Bioinformatics Service Unit, University of Haifa, Haifa, Israel; CorpoGen

**Keywords:** microbial fuel cells, community assembly, electroactive bacterial consortia, microbial ecology

## Abstract

Microbial fuel cells (MFCs) generate energy while aiding the biodegradation of waste through the activity of an electroactive mixed biofilm. Metabolic cooperation is essential for MFCs’ efficiency, especially during early colonization. Thus, examining specific ecological processes that drive the assembly of anode biofilms is highly important for shortening startup times and improving MFC performance, making this technology cost-effective and sustainable. Here, we use metagenomics to show that bioaugmentation of the anode surface with a taxonomically defined electroactive consortium, dominated by *Desulfuromonas*, resulted in an extremely rapid current density generation. Conversely, the untreated anode surface resulted in a highly stochastic and slower biofilm assembly. Remarkably, an efficient anode colonization process was obtained only if wastewater was added, leading to a nearly complete replacement of the bioaugmented community by Geobacter lovleyi. Although different approaches to improve MFC startup have been investigated, we propose that only the combination of anode bioaugmentation with wastewater inoculation can reduce stochasticity. Such an approach provides the conditions that support the growth of specific newly arriving species that positively support the fast establishment of a highly functional anode biofilm.

## Abstract (precis)

Mixed microbial communities play important roles in treating wastewater, in producing renewable energy, and in the bioremediation of pollutants in contaminated environments. While these processes are well known, especially the community structure and biodiversity, how to efficiently and robustly manage microbial community assembly remains unknown.

## INTRODUCTION

Microbial fuel cells (MFC) are electrochemical reactors that exploit microorganisms to promote the generation of renewable energy while treating different types of wastewater (1, 2). The operation of MFCs is based on the catalytic activity of an anaerobic electroactive biofilm that develops on the anode’s surface. Specific bacterial species (exoelectrogens) within this electroactive biofilm are capable of oxidizing the organic matter while releasing electrons via extracellular electron transport (EET) by using the anode as the terminal electron acceptor (3, 4). EET is a key metabolic process that facilitates organic matter degradation by anaerobic microbial communities in natural environments under specific redox gradients (5).

The electroactive biofilm formation process on the anode surface is predetermined during startup and critical for an efficient electricity-producing MFC (6, 7). The colonization of MFC anodes is generally regarded as a gradual process, accompanied by a shift in biodiversity, which consequently affects its electrical output even after achieving steady-state conditions (8–11). Hence, the anodic biofilm microbial composition and function have been considered vital components of the MFC performance, and the metabolic processes that characterize them have been intensively studied (11–19).

Shortening the MFC startup is one of the key steps that will allow MFC to fulfill their potential—utilizing wastewater as fuel. Different approaches for improving this step have thus been investigated, including chemical amendments to wastewater (20), chemical/physical anode surface pretreatment (21), applying an anodic positive poised potential (22), or directly inoculating the anode with preacclimated cultures (23). Although these microbe-centered methods have been somewhat efficient, typically, both exoelectrogens and nonexoelectrogens attach to the anode and develop into a biofilm. Consequently, establishment of the anodic biofilm, especially in the initial stages of colonization, involves both stochastic and specific selection processes that eventually dictate subsequent biofilm community composition at steady state and system performance (24, 25). While deterministic biofilm assembly in microbial electrochemical reactors often shows better reactor performance (24), it is still unclear how biofilm formation can be managed in such systems that would allow full control during early colonization. Such management schemes are applied by directing community structure and metabolic capacities in natural environments and bioreactors through bioaugmentation (26).

While most studies focus primarily on biofilm formation on clean surfaces (24, 25, 27, 28), our aim was to apply bioaugmentation to MFC anode surfaces in an attempt to significantly shorten biofilm formation startup times. We explored the effect of community assembly processes when the anode surface was precolonized (bioaugmented) by a defined electroactive consortium (designated EDC) on MFC startup. Due to selection and competition, such precolonization is expected to enhance deterministic factors relative to stochastic ones in the formation of the mature anode biofilm at steady state. We show that by augmenting the anode surface with EDC, the microbial performance was dramatically stimulated to produce an outstanding rapid current density compared with a slow startup when the anode surface was not applied as such. Importantly, optimal performance was observed by the augmented anode only when supplemented with wastewater, which points to key interspecies interactions that were dependent on specific newly arriving species provided through the wastewater.

## RESULTS

### Isolation of an enriched electroactive consortium and MFC bioaugmentation reduce time to stable current density production.

**FIG 1:**
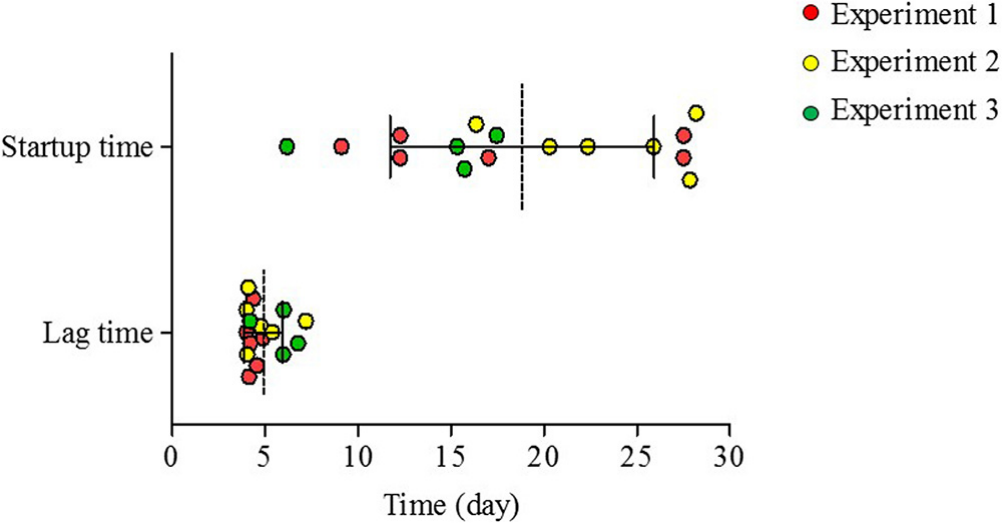
Scatter dot plot comparing lag times and startup times for replicate MFC reactors (*N* = 16) inoculated with diluted wastewater as the microbial source from three independent experiments. The horizontal lines indicate the means with error bars indicating standard deviations.

**FIG 2:**
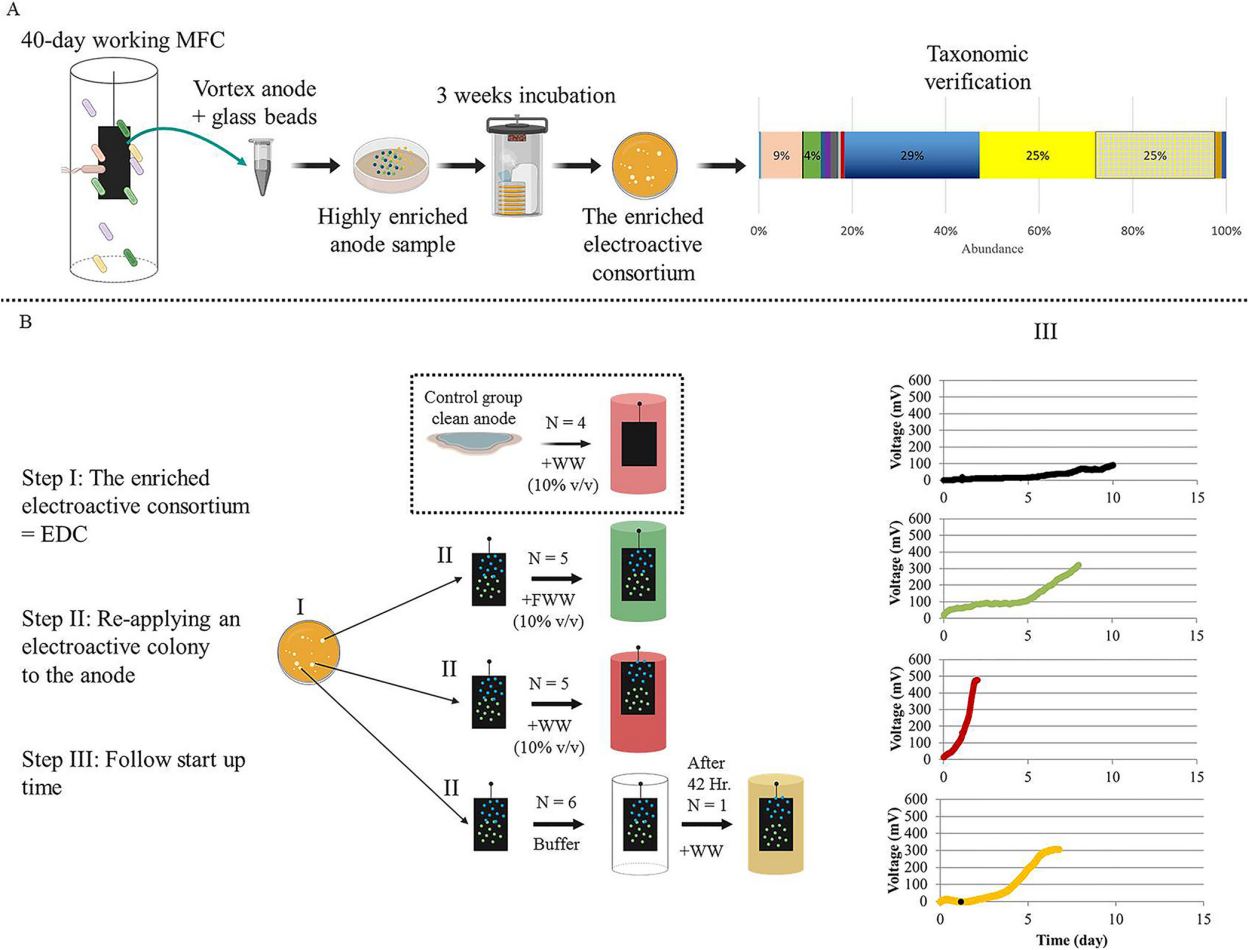
Schematic representation of experimental design and treatment groups. (A) Working scheme showing the way the electroactive enrichment (EDC) was obtained from an anode sample taken from a 40-day working MFC, incubated for several weeks, resulting in growth of colonies that were taxonomically verified by 16S amplicon sequencing to contain electroactive organisms. (B) Schematic representation of the experimental design. WW, wastewater; FWW, filtered wastewater; EDC, electroactive *Desulfuromonas* consortium. Created with BioRender (Toronto, Canada).

**FIG 3:**
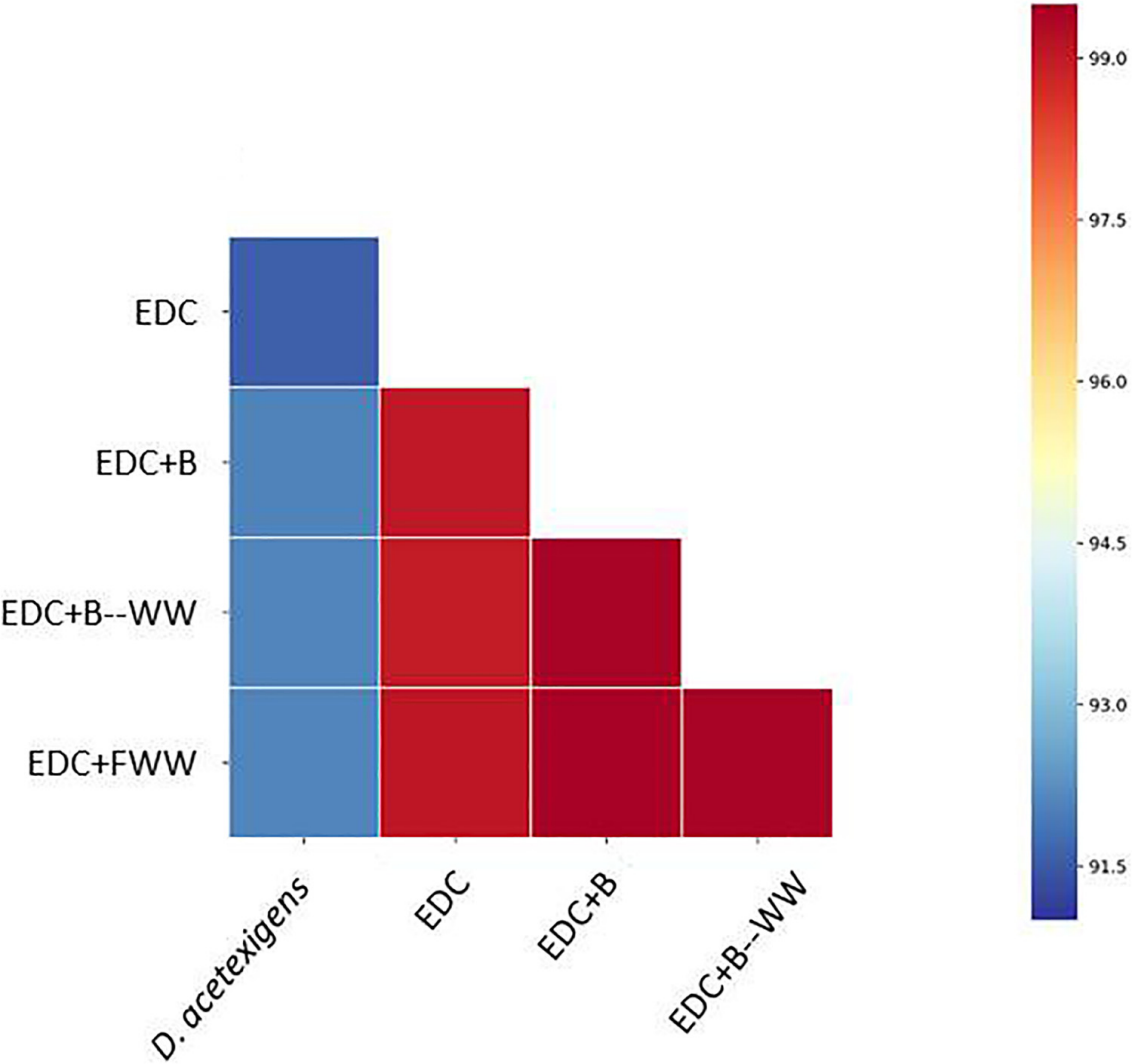
Heat map of pairwise average nucleotide identity (ANI) values for the genomes of *D. acetexigens* that were separately assembled from the four samples presented. *D. acetexigens* (53) was used as a reference genome.

Previous results have shown that the electroactive biofilm assembly process on clean anode surfaces, starting from the same inoculum, can be highly stochastic, leading to different startup times and community compositions (19). We repeated this on a larger scale, following 16 reactors inoculated with diluted wastewater and clean anode surfaces for as long as it took them to achieve stable current density production (defined as startup time) (Fig. 1). Startup time again showed high variation between cells (18.83 ± 7.11 days), while lag times (the time to achieve primary current density production) tended to be more similar among the 16 reactors (4.92 ± 1.04 days) (Fig. 1). Therefore, we hypothesized that in order to reduce the high stochasticity characterizing early biofilm assembly, deterministic factors should be enhanced by precolonizing the anode surface with a defined microbial community composed primarily of electroactive species. We thus augmented MFC anodes by directly applying a microbial electroactive consortium isolated from a working anode (Fig. 2A). This consortium (EDC) was dominated by two or more *Desulfuromonas* strains that were distantly related to Desulfuromonas acetexigens (average nucleotide identity [ANI] of 91.5 to 92.1%) (Fig. 3), but relatively similar to one another (ANI of 99 to 99.4%) in all EDC batches, indicating that similar strains of *Desulfuromonas* colonized all replicate anode surfaces (Fig. 3).

**FIG 4:**
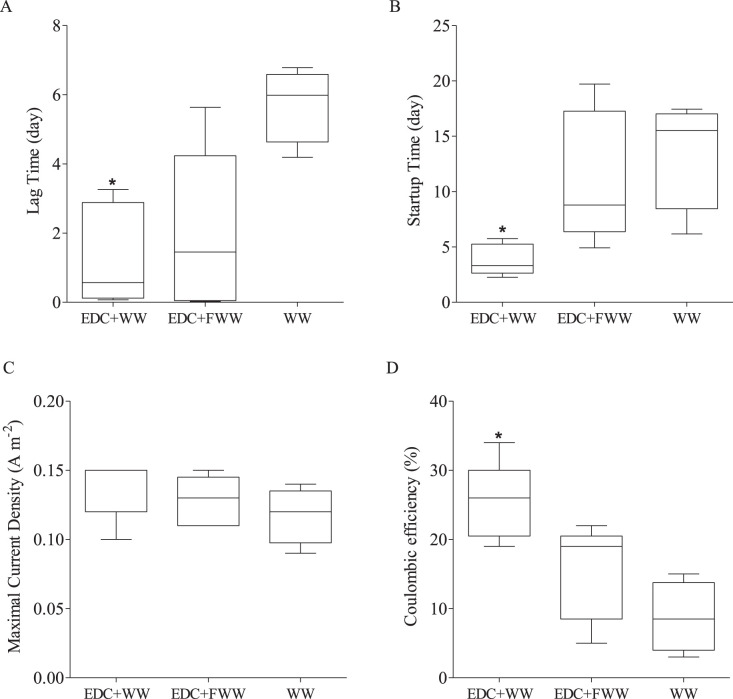
Lag times (A), startup times (B), maximal current densities (C), and coulombic efficiency (D) of the MFC reactors of three treatment groups: EDC+WW (*N* = 5), EDC+FWW (*N* = 5), and WW (*N* = 4). *, *P* < 0.05 (Kruskal-Wallis test).

When anodes to which EDC was applied were placed in an MFC-containing bicarbonate buffer and acetate as a carbon source (*N* = 6), current density increased rapidly but quickly dropped to zero 48 to 72 h after (see Fig. S1A in the supplemental material). Recovery of current density was observed when the MFCs’ bulk liquid was switched to either dilute wastewater or dilute 0.2-μm-filtered wastewater (10% [vol/vol]) immediately after that drop. To investigate this phenomenon, three different treatments were compared, as illustrated in Fig. 2B. The first group of MFCs (*N* = 5) was inoculated with diluted filtered wastewater (EDC+FWW), and a second group (*N* = 5) was inoculated similarly with diluted wastewater including their entire microbial diversity (EDC+WW). The anode surfaces of both of these groups were pretreated with EDC, as described in Materials and Methods. A control group of MFCs (*N* = 4) was inoculated with wastewater without applying EDC to the anode (i.e., a clean anode surface). All solutions were supplemented with acetate. This experiment demonstrated that both lag time and startup time were significantly lower in bioreactors that had the EDC-augmented anodes with diluted wastewater than those with control MFCs (Fig. 4A and B) (Kruskal-Wallis test; *P* < 0.05). Notably, mean lag time and startup times of EDC-augmented anodes with filtered wastewater showed a moderate improvement (nonsignificant Kruskal-Wallis test; *P* > 0.05), in agreement with a recent study that found that the performance of bioelectrochemical reactors declined as biofilm diversity decreased (24). In contrast, no difference was observed among the three treatments for maximal current density obtained (Fig. 4C), yet coulombic efficiency was significantly higher in bioreactors that had the EDC-augmented anodes with diluted wastewater that those with control MFCs (Fig. 4D).

10.1128/mBio.03629-20.1 FIG S1 (A) Electrochemical behavior of the MFCs indicating the time at which the bulk liquid was changed. (B) Electrochemical behavior of control MFC reactor inoculated with filtered wastewater and a clean anode. Download FIG S1, PDF file, 0.2 MB. Copyright © 2021 Yanuka-Golub et al. 2021 Yanuka-Golub et al. https://creativecommons.org/licenses/by/4.0/ This content is distributed under the terms of the Creative Commons Attribution 4.0 International license.

The filtration treatment did not affect the chemical composition of essential components in wastewater, with the exception of organic nitrogen, which was 2-fold reduced in filtered wastewater (see Fig. S2). 16S rRNA gene amplicon sequencing showed that, as expected, filtered wastewater used for inoculation (i.e., day 0) lacked many of the bacteria present in unfiltered wastewater and consequently had lower diversity (Shannon of 6.2 ± 0.2 versus 9.7 ± 0.03 and Faith’s phylogenetic diversity [PD] of 10.1 ± 2.02 versus 59.5 ± 1.6, respectively). The filtered wastewater community comprised several small cell taxa that could potentially pass through the 0.22-μm filter (see Fig. S3 and S4). To examine the contribution of these species to electroactive biofilm formation, a different control MFC group, inoculated with filtered wastewater with a clean anode (henceforth, blank control) was tested and showed a slight increase in current density over time (Fig. S1B), indicating that this inoculum has some limited electrogenic biofilm-forming potential. Therefore, we next asked which factor(s) led to such a strong synergy between the wastewater “source community” composition and the precolonized anode community, which together achieved a dramatically higher functional anode biofilm.

10.1128/mBio.03629-20.2 FIG S2 (A) Biochemical oxygen demand (BOD) and chemical oxygen demand (COD) analysis results for the sterile bicarbonate buffer (BCM), wastewater, and filtered wastewater (10% [vol/vol]) used for inoculation of MFCs. (B) Nitrogen and phosphate concentrations measured for the sterile BCM, filtered wastewater (10% [vol/vol]), and wastewater (10% [vol/vol]). (C) Annual wastewater concentrations of BOD, COD, and total suspended solids (TSS) reported by the wastewater treatment plant. (D) Taxonomic composition (phylum level) of wastewater samples taken for experiments 1 to 3. (E) Taxonomic composition (*Proteobacteria* class level) of wastewater samples taken for experiments 1 to 3. (F) Alpha and beta diversity for the wastewater samples taken for inoculation of experiments 1 to 3. Download FIG S2, PDF file, 0.4 MB. Copyright © 2021 Yanuka-Golub et al. 2021 Yanuka-Golub et al. https://creativecommons.org/licenses/by/4.0/ This content is distributed under the terms of the Creative Commons Attribution 4.0 International license.

10.1128/mBio.03629-20.3 FIG S3 (A) Distribution of taxa in the unfiltered wastewater solution used for inoculating the MFCs. (B) Distribution of taxa in the filtered wastewater solution used for inoculating the MFCs. Bacterial relative abundances are shown for both plots at the family level via 16S rRNA gene sequencing. Both solutions were supplemented with acetate and diluted in BCM. Download FIG S3, PDF file, 0.2 MB. Copyright © 2021 Yanuka-Golub et al. 2021 Yanuka-Golub et al. https://creativecommons.org/licenses/by/4.0/ This content is distributed under the terms of the Creative Commons Attribution 4.0 International license.

10.1128/mBio.03629-20.4 FIG S4 Shannon and phylogenetic diversity indices of the filtered and unfiltered wastewater solutions used for MFC inoculation. Download FIG S4, PDF file, 0.08 MB. Copyright © 2021 Yanuka-Golub et al. 2021 Yanuka-Golub et al. https://creativecommons.org/licenses/by/4.0/ This content is distributed under the terms of the Creative Commons Attribution 4.0 International license.

### Predictable development of a highly functional anode-colonizing biofilm requires specific wastewater-borne bacteria.

Potentially, augmentation of the anode with an electroactive consortium should not only shorten startup time but also result in more predictable MFC communities similar to one another. To compare the microbial community composition of each of the precolonized anode surfaces (EDC+FWW and EDC+WW treatment groups) to those of nonpretreated anodes (WW), the anodic microbial communities of all reactors were collected and compared once stable current density was obtained (defined as steady state). Additional anode samples were taken at different time points from bioreactors that initially contained only bicarbonate buffer with acetate and EDC-augmented anodes (*N* = 6) after the current density drop (see above) at 42 h, some of which were supplemented with wastewater (Fig. 2B). The planktonic community in each of the treatment groups was also sampled at different time points (total, 48 samples).

**FIG 5:**
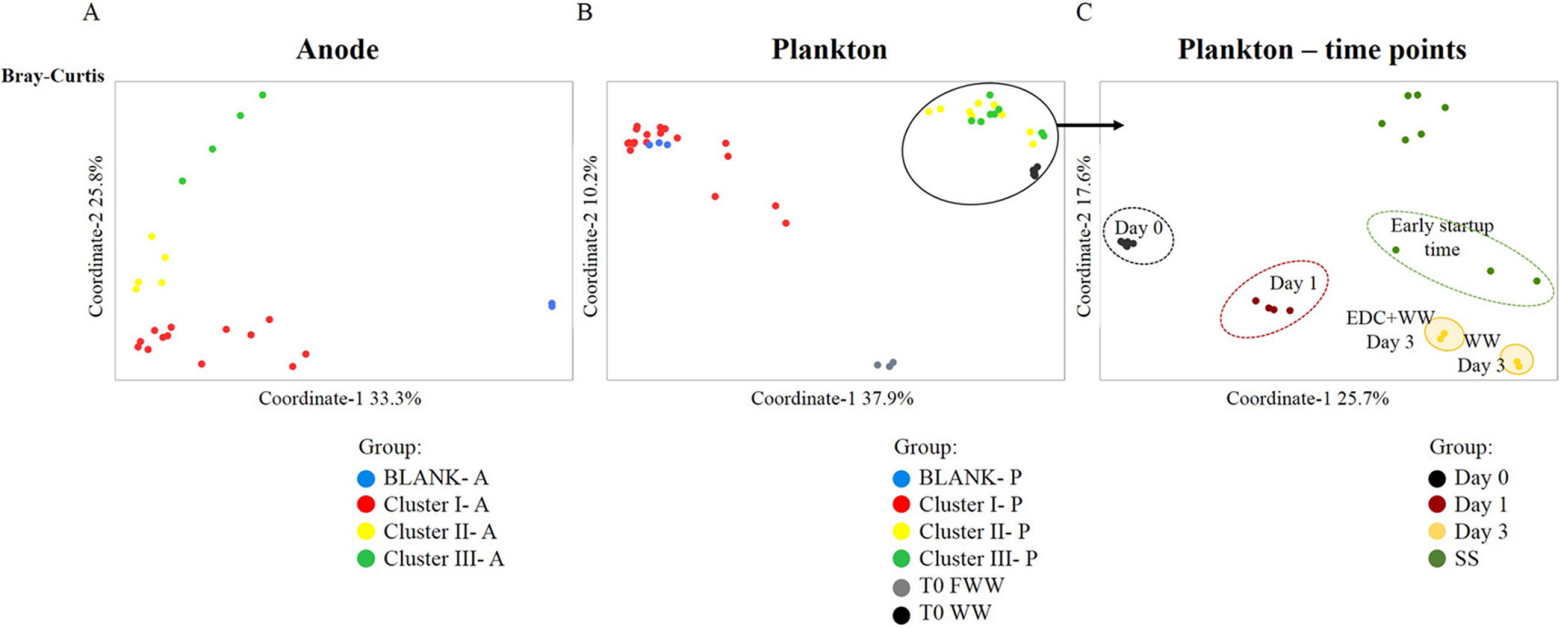
Principal-coordinate analysis (OTU level) based on Bray-Curtis distances of the anode-biofilm communities (blank, control of filtered wastewater with a clean anode) (A), plankton communities (B), and plankton communities of only clusters II and III at specific time points (C): day 0 at which wastewater was supplemented with the bioaugmented anode; Day 1 and Day 3 denote sampling time point; SS, steady state, the time at which stable maximal current density was achieved, which varied for each MFC, depending on when the last electrochemical cycle terminated.

Overall, the communities could be assigned to four clusters (Table 1) according to their composition regardless of the distance measure used (Bray-Curtis, weighted or unweighted UniFrac) (Fig. 5; see also Fig. S5). Cluster I contained samples that were similar in taxonomic composition to the EDC: EDC plus bicarbonate buffer (EDC+B), EDC+FWW, and EDC+B switched to wastewater (EDC+B–WW). Cluster II comprised samples that had a statistically significant positive effect on both lag time and startup time (EDC+WW) (Fig. 4A and B) relative to nonbioaugmented MFCs. Cluster III included clean anodes that were not pretreated with EDC (i.e., WW), and the cluster IV comprised blank controls (MFCs inoculated with filtered wastewater and a clean anode, i.e., blank control).

**TABLE 1:** Summary of treatment groups employed in this study

Treatment group	Description	Supplemented liquid in MFC at day 0	Anode bioaugmented with EDC	No. of replicates	Cluster^*a*^
WW	Control group for testing startup time without the direct application of EDC	Diluted wastewater 10% (vol/vol) plus acetate	No	4	III
EDC+B	Anode surface bioaugmented with EDC and supplemented with BCM^*b*^	BCM plus acetate	Yes	6	I
EDC+B–WW	After 1 day, one of the EDC+B anodes was switched to MFC-containing buffer	After one day as EDC+B, switched to diluted wastewater 10% (vol/vol) plus acetate	Yes	1	I
EDC+WW	Anode surface bioaugmented with EDC and supplemented with wastewater	Diluted wastewater 10% (vol/vol) plus acetate	Yes	5	II
EDC+FWW	Anode surface bioaugmented with EDC and supplemented with filtered wastewater	Diluted filtered wastewater 10% (vol/vol) plus acetate	Yes	5	I
Blank	Filtered wastewater with a clean anode; control	Diluted filtered wastewater 10% (vol/vol) plus acetate	No	2	IV

a According to Fig. 5.

b BCM, bicarbonate buffer.

10.1128/mBio.03629-20.5 FIG S5 Principal-coordinate analysis (OTU level) based on UniFrac (weighted or unweighted) or Bray-Curtis distances (each PCoA uses one measure) of the anode-biofilm communities (blank, control of filtered wastewater with a clean anode; cluster I, EDC, EDC+B, EDC+FWW, and EDC+B–WW; cluster II, EDC+WW; xluster III, control, MFC supplemented with wastewater [WW] and clean anode) (A), plankton communities (B), and plankton communities of only clusters II and III at specific time points (C): day 0 at which wastewater was supplemented with the bioaugmented anode; D1, day 1 after inoculation; D3, day 3; SS, steady state, the time at which stable maximal current density was achieved, which varied for each MFC depending on when the last electrochemical cycle terminated. Download FIG S5, PDF file, 0.5 MB. Copyright © 2021 Yanuka-Golub et al. 2021 Yanuka-Golub et al. https://creativecommons.org/licenses/by/4.0/ This content is distributed under the terms of the Creative Commons Attribution 4.0 International license.

Analysis of similarity (ANOSIM) of anode communities indicated that cluster I was significantly different from clusters II and III, but the clusters differed to a larger degree in the planktonic compared to the anodic communities (see Table S1). On the other hand, while anode samples of clusters II and III were somewhat similar according to the weighted distances, the planktonic samples of these two groups clustered exclusively together according to all distance measures (Fig. 5B; Table S1). This may suggest that the difference between cluster I and clusters II/III was caused by the emergence of specific taxonomic groups originating from the wastewater “source community” as well as specific interspecies interactions that may have occurred between those source communities and the communities that already precolonized the anode by applying EDC on its surface.

10.1128/mBio.03629-20.10 TABLE S1 ANOSIM results of PCoA (for Fig. 5) and DSeq full data results (for Fig. 7). Download Table S1, XLSX file, 0.03 MB. Copyright © 2021 Yanuka-Golub et al. 2021 Yanuka-Golub et al. https://creativecommons.org/licenses/by/4.0/ This content is distributed under the terms of the Creative Commons Attribution 4.0 International license.

**FIG 6:**
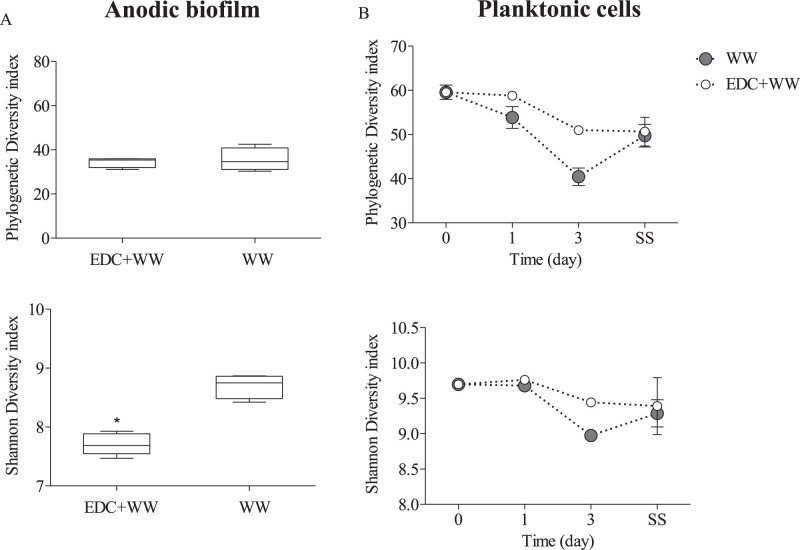
(A) Alpha diversity for anode-attached communities in the MFCs of two groups: augmented anodes (EDC+WW) and control nonaugmented anodes (WW). (B) Temporal dynamics of the planktonic communities of the same two groups. The last time point is SS, indicating the time at which steady-state current production was achieved. For the WW group, this time was significantly longer than for EDC+WW, as explained in the main text.

It was intriguing that although both anodic and planktonic communities of cluster II and cluster III (EDC+WW and WW, respectively) were taxonomically similar, each system exhibited a substantially different startup time (Fig. 4A and B). This fundamental difference was hypothesized to occur already at early time points due to specific interactions between the planktonic community and EDC precolonizers on the anode surface of EDC+WW. Specific planktonic community assembly processes occurred at very early time points in MFCs pretreated with EDC, which subsequently affected their corresponding anodic community structure and startup time. Exclusively, while phylogenetic diversity index was similar, the Shannon diversity index of bacterial communities on the clean anode samples was significantly higher than those of the EDC-precolonized anode surface (*P* < 0.05, *t* test) (Fig. 6A). The planktonic community dynamics changed over time, but only on day 3, beta-diversity analyses showed differences between the two treatments (Fig. 5C). This observation was in agreement with the differences in alpha diversity change with time of the planktonic community (Fig. 6B). Remarkably, the time the bioreactors (EDC augmented) took to reach maximal current density was between 2.2 and 5.7 days (3.8 ± 1.4 days) (Fig. 4B), coinciding with the observed planktonic community divergence on day 3. Overall, these results indicate that specific planktonic community assembly processes occurred at very early time points in MFCs pretreated with EDC, which subsequently affected their corresponding anodic community structure and startup time. Finally, once steady-state conditions were achieved, the planktonic communities of both treatments converged to similar bacterial communities (Fig. 6B).

**FIG 7:**
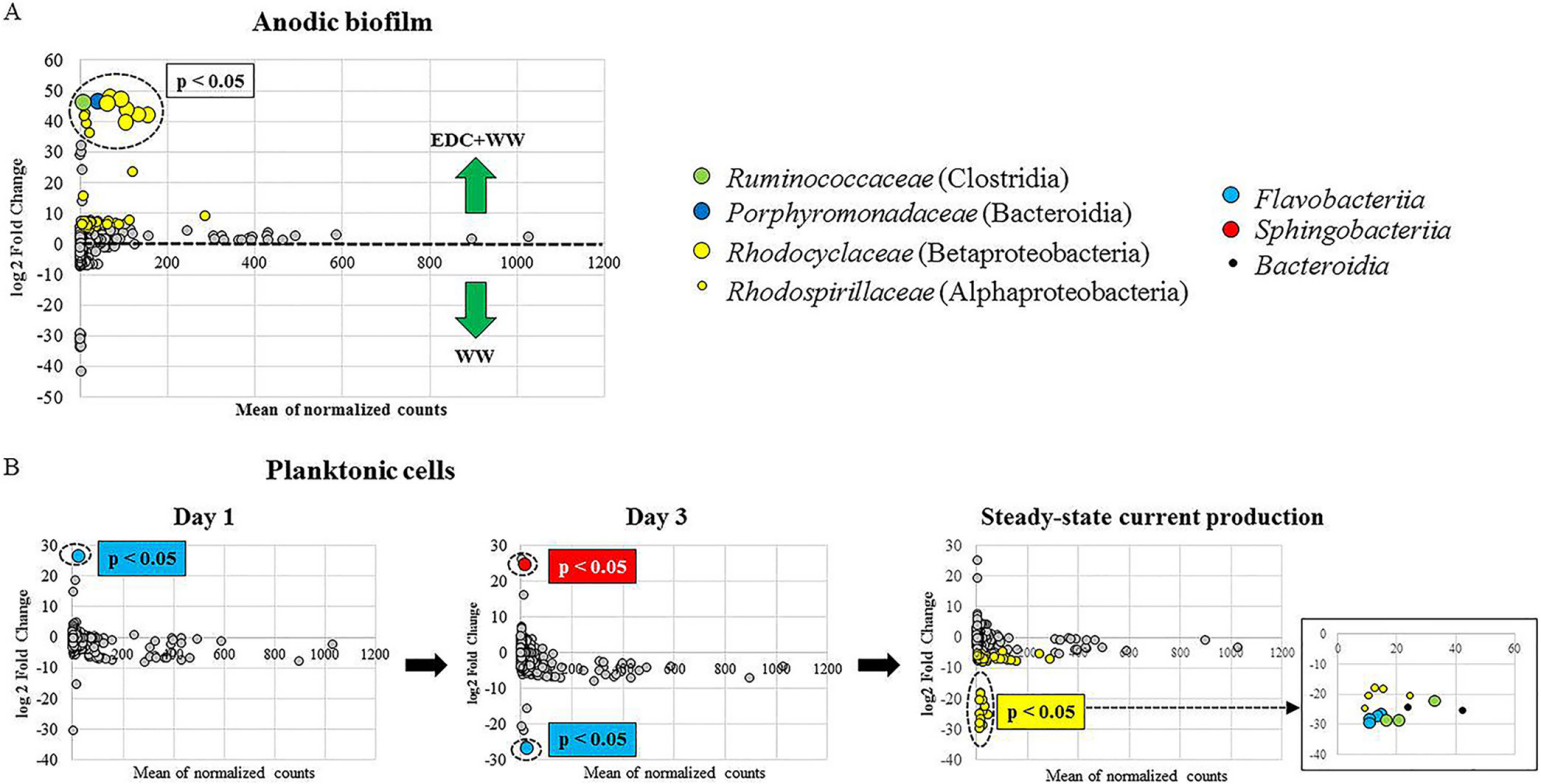
Differentially abundant bacterial members of the anode and planktonic communities of MFCs. MA plots depicts the log_2_ fold change of OTUs significantly enriched or depleted (FDR < 0.05) in anode-attached communities (A) and planktonic communities (B).

To explain the observed differences between EDC+WW and WW samples, DESeq2 was used to identify differentially abundant amplicon sequences in the EDC+WW group (positive fold change) compared to those in the WW group (negative fold change indicates operational taxonomic units [OTUs] enriched in WW). Anode and planktonic samples were analyzed separately. At the anode, 49 OTUs were significantly enriched in samples EDC+WW compared to that in WW (false-discovery rate [FDR] < 0.01) (Fig. 7A; see also Table S1). Comparing the planktonic sequences of day 1 showed that only one OTU was significantly enriched in EDC+WW (member of *Flavobacteriia*); subsequently, on day 3, this same OTU became more abundant in the WW planktonic community and was replaced by a different OTU (member of *Sphingobacteriia*) in the EDC+WW planktonic community (Fig. 7B). In steady state, 63 OTUs were significantly enriched or depleted in the EDC+WW planktonic community (Fig. 7B; Table S1), in which Alphaproteobacteria, Clostridia, and Flavobacteriia were the most abundant groups.

**FIG 8:**
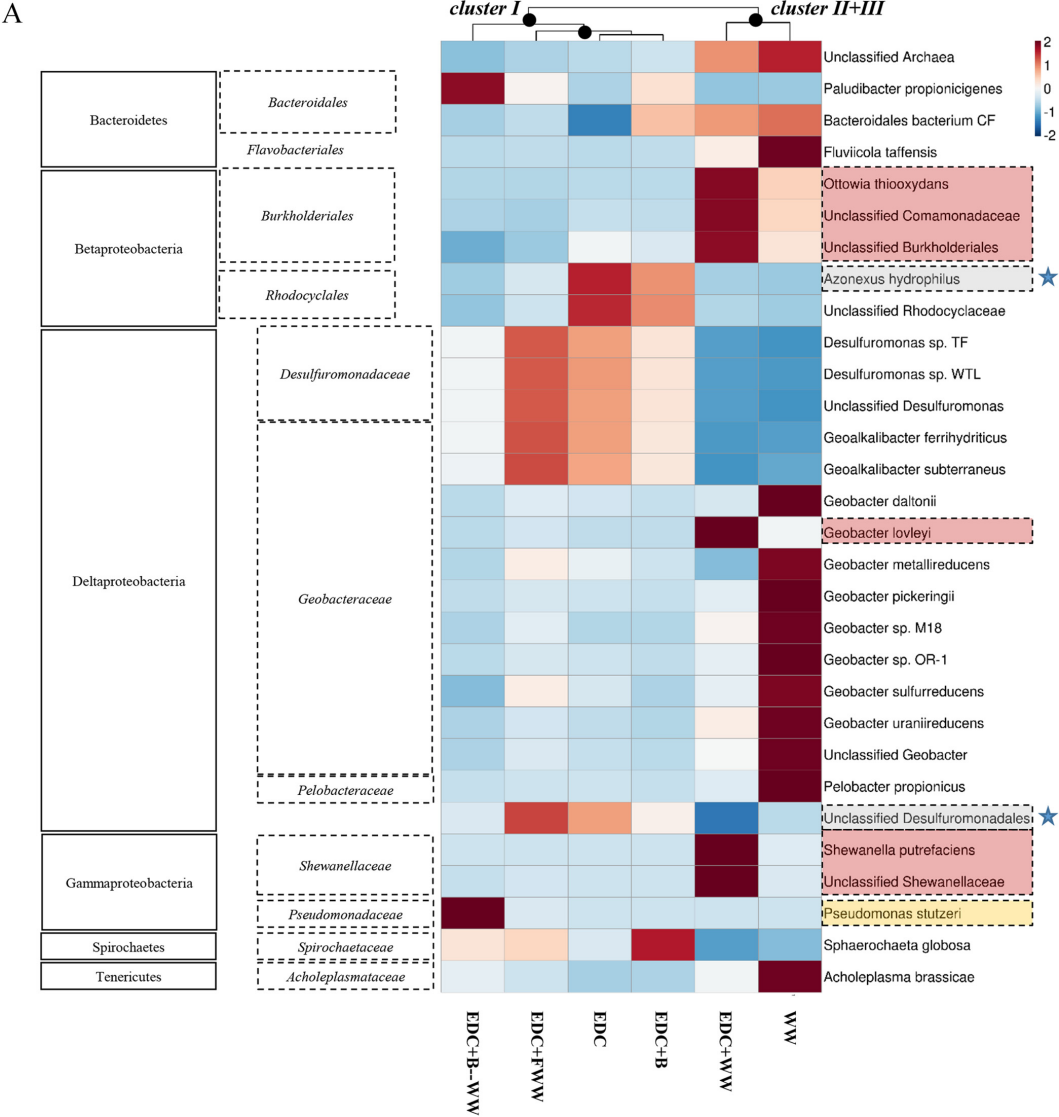

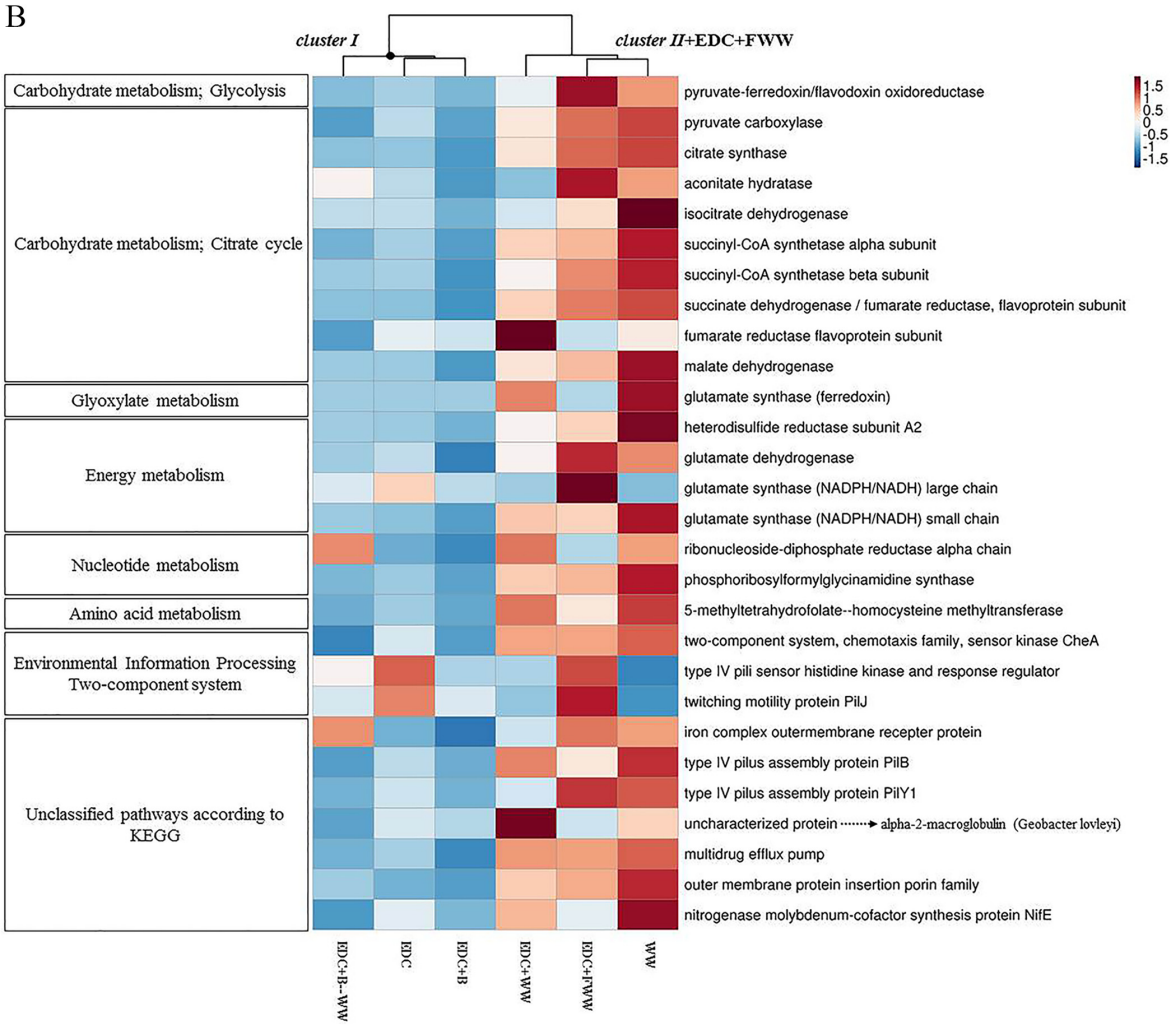
Heat maps of bacterial taxonomic compositions (A) and KEGG ortholog functions (B). Both heat maps are based on shotgun metagenomics, and clustering of the samples was computed using the Bray-Curtis dissimilarity index. In panel A, the blue star shape is for the two most abundant species found in the EDC enrichment according to 16S amplicon and metagenomics sequencing. Species marked with a red rectangle were found most abundant on the EDC+WW anode surface.

To obtain a higher taxonomic resolution of the established anodic biofilm and the differences between clusters I to III, as well as discover potential functional genes that they harbor, six representative anode samples were sequenced via shotgun metagenomics, and the obtained reads were assigned taxonomic and functional classifications. Indeed, metagenomics data of the six representative anode samples were in agreement with the 16S amplicon sequencing with respect to the most differentially abundant taxonomic groups identified according to DESeq analysis (see Fig. S6) as well as the strong taxonomic association between the EDC+WW and WW anode samples (see Fig. S7). Taxonomic observations of shotgun metagenomics data revealed important differences between clusters I and II (Fig. 8A). First, the most abundant family belonging to Deltaproteobacteria in cluster I was Desulfuromonadaceae, originating from the bioaugmenting consortium, as described above. However, the most abundant family in cluster II was *Geobacteraceae*, which reached 39% of the total community. In sample EDC+WW, Geobacter lovleyi was the single species that dominated (31%); in WW without an EDC pretreatment, other Geobacteraceae species were more abundant than in other samples.

10.1128/mBio.03629-20.6 FIG S6 Relative abundance of key taxonomic groups according to 16S rRNA gene amplicon and shotgun metagenomics sequencing. Download FIG S6, PDF file, 0.2 MB. Copyright © 2021 Yanuka-Golub et al. 2021 Yanuka-Golub et al. https://creativecommons.org/licenses/by/4.0/ This content is distributed under the terms of the Creative Commons Attribution 4.0 International license.

10.1128/mBio.03629-20.7 FIG S7 Cluster analysis of the six samples sequenced via shotgun metagenomics for the KEGG ortholog functions and taxonomic classifications. Clustering was computed using the Bray-Curtis dissimilarity index. Download FIG S7, PDF file, 0.01 MB. Copyright © 2021 Yanuka-Golub et al. 2021 Yanuka-Golub et al. https://creativecommons.org/licenses/by/4.0/ This content is distributed under the terms of the Creative Commons Attribution 4.0 International license.

Azonexus hydrophilus was the most dominant species in cluster I aside from members of the Desulfuromonadaceae. In samples WW, EDC+WW, and EDC+B–WW, all of which had wastewater supplementation, relative abundance of *A. hydrophilus* was <1%. The relative abundance of Gammaproteobacteria was the highest in sample EDC+B–WW, in which Pseudomonas stutzeri was dominant. On the other hand, *Shewanella* sp., a well-known electroactive organism belonging to Gammaproteobacteria, was mainly enriched in sample EDC+WW.

### The highly functional anode-colonizing community was linked to chemotaxis regulation, type IV pilus assembly, and colonization proteins.

To better understand how the taxonomic differences correspond to functional differences, we compared the abundances of KEGG ortholog functions (KOs) in the six anode biofilm communities. Overall, the six samples had relatively similar functional profiles with two clusters splitting at ∼75% similarity (based on Bray-Curtis dissimilarity index) (Fig. S7). Notably, while the anode sample EDC+FWW was related in terms of predicted functions to samples WW and EDC+WW, taxonomically it clustered with EDC and EDC+B (Fig. 8A and B). Higher numbers of proteins associated with motility, chemotaxis, signal transduction, membrane transport, and carbohydrate metabolism were observed in the anodic biofilms belonging to cluster II and sample EDC+FWW. Sample EDC+WW had a unique functional profile, explicitly resulting from the coupling of wastewater and the precolonized anode (Fig. 8B). Six protein-coding genes were found to be specifically associated with samples EDC+WW and WW and not EDC+FWW (Fig. 8B). Four were associated with KEGG metabolic pathways (fumarate reductase, glutamate synthase, *nifE*, and homocysteine methyltransferase). Fumarate reductase, an important enzyme for anaerobic respiration (29), was primarily harbored by *A. hydrophilus* in EDC and *G. lovleyi* in EDC+WW and had the highest abundance in EDC+WW. This probably indicates the specific contribution of *G. lovleyi* to acetate oxidation through central metabolic pathways, leading to an efficient EET that eventually benefits the entire anodic biofilm. Other metabolic genes that were *Geobacter*-associated were those encoding glutamate synthase, an iron-sulfur flavoprotein that plays a key role in ammonia assimilation (30), and 5-methyltetrahydrofolate-homocysteine methyltransferase, previously detected as one of the most highly expressed genes on closed circuit electrode surfaces (31). The nitrogenase molybdenum-cofactor biosynthesis protein NifE has a critical role in nitrogen fixation (32). NifE was primarily harbored by *Geobacter* in EDC+WW and WW anode samples and by *Desulfuromonas* and *A. hydrophilus* in EDC, EDC+B, and EDC+FWW. Additional differential functions were type IV pilus assembly protein PilB and an uncharacterized protein homologous to alpha-2-macroglobulin of *G. lovleyi*, which are likely to be important in surface colonization (33, 34).

## DISCUSSION

Microbial community assembly on surfaces has long been of great interest for a wide range of community ecology disciplines, including plants, algae, and microorganisms (35). In microbial systems, most studies are associated particularly with the colonization of new surfaces, according to the model suggested by Jackson (35) which describes the changes in community properties that occur during bacterial biofilm succession starting from the very early stages until the biofilm is well established. Conversely, in this study, we explored biofilm assembly on a precolonized surface, which is especially relevant when applying bioaugmentation for *in situ* bioremediation of natural environments (36) or wastewater treatment (37). In groundwater, it has been shown that the composition of sediment-attached communities can differ substantially compared to that of suspended planktonic communities (28, 38); therefore, the use of MFC reactors as a model system allows full control of the environmental conditions and easy differentiation between surface-attached (i.e., anodic biofilm) and suspended communities (24, 25). The importance of analyzing the suspended community (planktonic cells) dynamics lies in the two potential sources of the planktonic bacteria. The first is the wastewater in the MFC that provide a microbial source (i.e., a pool of newly arriving species), and the second is cells that are released from the anodic biofilm into the bulk liquid. The latter implies that the planktonic community can potentially reflect the anodic biofilm assembly (19), without harming the anode. Following planktonic community dynamics is especially important during the very early stages when the tipping point between deterministic and stochastic processes can substantially affect the interaction between the new-arriving species and the founders (39). Furthermore, MFCs’ current density serves as a biosignal directly associated with the anodic microbial community performance and biofilm formation stages with time.

Direct application of an enriched consortium combined with wastewater substantially reduced startup times to 3.8 days from an average of 18 days observed for 16 empty anode surfaces. Interestingly, the immediate current density produced was not stable unless the anode surface was bioaugmented and also coinoculated with either filtered wastewater or wastewater. Although other studies reached similar reduced startup times by applying an anodic positive poised potential (22) or different biofilm acclimatization procedures (23, 40), our study systematically demonstrated the importance of both the enriched consortium used to acclimate the anode surface and the wastewater as a source of critical species that sustain the anode biofilm formation process, as discussed below.

Filtered wastewater had a reduced effect on startup time relative to that of nonfiltered wastewater (Fig. 4), in agreement with a recent study that found that the performance of bioelectrochemical reactors declined as biofilm diversity decreased (24). The taxonomic composition of filtered wastewater anodes resembled EDC and was substantially different from that of nonfiltered wastewater anodes (Fig. 5 and 8A). This implies that a successful anode colonization process (of sample EDC+WW), which led to an extremely fast and robust startup time, involved a large shift in the anode-associated founder community. A particularly interesting switch was observed between the two main exoelectrogenic species, *Desulfuromonas* spp. and Geobacter lovleyi, which suggests a specific interaction that could exist between these closely related species in other systems, for example, sediment-attached communities in subsurface environments. Furthermore, the fact that this taxonomic shift did not occur in samples EDC+B, EDC+FWW, and EDC+B–WW indicates that these samples had a relatively similar anode biofilm composition, further supporting the importance of the addition of the complete microbial source (wastewater instead of filtered wastewater) to rapidly achieve a high-functioning electroactive community on the anode surface. Since Geobacteraceae and Desulfuromonadaceae are phylogenetically related (41), occupy similar niches. and use the same carbon sources and electron acceptors (both in engineered and natural systems), it is reasonable to hypothesize that when seeded at time zero, these groups (as planktonic free-living cells in the bulk liquid) compete for the same resources (electron donors and available space to attach to the anode, colonize, and transfer electrons to it).

Anode biofilm compositions of the two groups EDC+WW and WW under steady-state conditions were highly similar, yet MFCs exhibited substantially different startup times. This fundamental difference was hypothesized to occur already at early time points due to specific interactions between the planktonic community and EDC precolonizers on the anode surface of EDC+WW. Further analysis revealed differentially abundant OTUs belonging to Flavobacteriia and Sphingobacteriia on days 1 and 3, respectively. On day 3, corresponding to the average startup time of EDC+WW reactors, the only OTU that became differentially abundant in EDC+WW relative to the control planktonic communities belonged to Sphingobacteriia. This finding is consistent with the functional role attributed to Flavobacteriia and Sphingobacteriia, both of which were found to represent key members in the formation and functioning of biofilms in diverse environments (42–44), probably due to their ability to degrade diverse complex organic material and their superior attachment ability. Furthermore, Sphingobacteriia were previously reported in bioelectrochemical devices both at the plankton fraction and anodic biofilm as homoacetogens or fermenters of complex organic substances that support electrogenic species within the biofilm through syntrophic interactions (45). Syntrophic interactions between electroactive and nonelectroactive bacteria in the anodes of microbial electrochemical cells have been a widely studied topic for more than a decade (46). Such coordinated interactions between different functional groups have been shown to increase efficiency of anaerobic systems. For example, Parameswaran et al. (47) showed that microbial electrolysis cells fed with H_2_ as the sole electron donor positively affected the syntrophic interaction of homoacetogens with anode-electroactive species (*Geobacter*), which led to current densities comparable to those obtained by acetate-fed biofilms. While most studies focused on such interactions during steady-state conditions, aiming to improve system efficiency after the anode has already matured, here we show that syntrophic interactions are important during the early anode acclimation period as well. This was evident when the anode surface was pretreated with EDC, leading to specific selection processes.

Additionally, nitrogen fixation-related genes (*nifE* and *nifH*) were mostly abundant with EDC, EDC+WW, and WW anode biofilms. While *nifE* in the latter two groups was affiliated with *Geobacteraceae* family members, in the former ones it was associated with *A*. *hydrophilus* and *D. acetexigens* (see Fig. S8 in the supplemental material). Overall, the combined results from amplicon sequencing with the taxonomic and functional classifications obtained by shotgun metagenomics illustrate that only when supplemented with nonfiltered wastewater, a replacement of the founder species, EDC, (as well as key functional traits) on the anode biofilm occurred (i.e., a nearly complete turnover). Interestingly, even though the filtered wastewater did contain specific species that allowed an improved startup time relative to that for control MFCs, they did not eventually replace any of the founder species of EDC at steady state.

10.1128/mBio.03629-20.8 FIG S8 Taxonomic affiliations of four KEGG ortholog functions. Download FIG S8, PDF file, 0.01 MB. Copyright © 2021 Yanuka-Golub et al. 2021 Yanuka-Golub et al. https://creativecommons.org/licenses/by/4.0/ This content is distributed under the terms of the Creative Commons Attribution 4.0 International license.

In this study, two different mature communities had very similar taxonomic and functional configurations, yet the early stage assembly processes substantially differed between them, resulting in different startup times. Thus, we show for the first time that precolonizing the anode surface with a defined electroactive consortium led to a strong synergy between the planktonic “source community” and the precolonized anode community. Essentially, we suggest that the precolonized anode created specific niches that became available and were quickly occupied by the newly arriving species, especially key members that are biofilm formers (i.e., Flavobacteriia and Sphingobacteriia) that aided the electrogenic bacteria in establishing a highly functional anode biofilm via syntrophic interactions. This enhanced niche-based process dramatically stimulated the MFC performance and thus offer a new approach for minimizing the system’s stochasticity and a better way to select for the most efficient microorganisms for environmental biotechnology.

## MATERIALS AND METHODS

### MFC setup, experimental design, and operation.

Single-chamber air-cathode MFCs were used for the enrichment of electroactive bacteria and suspended-cell (plankton) communities, as previously described (19). The experimental setup aimed to directly apply on the anode surface an electroactive consortium, EDC, composed of >50% *Desulfuromonas* spp. enriched from an active MFC (Fig. 2A). Before comparing between processes governing microbial community assembly and succession on clean anodes to those preapplied with EDC, three independent experiments (total *N* = 16) were conducted to determine average startup rates of replicate MFC reactors inoculated using fresh diluted wastewater (10% [vol/vol]) sample as the microbial source (wastewater was sampled the day prior to experiment initiation). Therefore, each experiment (1–3) had a different starting microbial composition, which was characterized by 16S rRNA amplicon sequencing (see Fig. S2 in the supplemental material). Municipal wastewater was collected from the anoxic zone of an aeration tank (Kolchai HaSharon WWTP, Hof Hasharon Regional Council, Israel), and diluted with bicarbonate-buffered medium (30 mM BCM), as previously described (19). Characteristics of the wastewater used are shown in Fig. S2.

The second step of the experiment was to compare startup times of MFCs preinoculated with EDC supplemented with wastewater (*N* = 5) and filtered wastewater (0.2 μm; *N* = 5) (Fig. 2B). All reactors were supplemented with acetate (20 mM) as a carbon source, in addition to the wastewater, in order to provide a sufficient amount of organic carbon for all treatment groups.

### Wastewater filtration.

Raw wastewater was first filtered through Whatman grade 1 qualitative filtration paper to remove large particles and then through a 0.22-μm filter (Stericup-GP, 0.22-μm polyethersulfone; Merck) for the removal of the majority of microorganisms present in the sample.

### Applying the enriched *Desulfuromonas* consortium to the anode surface.

We specifically chose to sample an anode from an MFC that had been operating for 40 days (reached steady-state current density generation). Based on previous results (19), the anode was assumed to be colonized by members of the *Desulfuromonadaceae* family, and this was later verified by 16S rRNA gene amplicon sequencing (data not shown). Anode samples were plated on bicarbonate sodium fumarate plates and incubated anaerobically until light-orange-colored colonies appeared after 3 weeks (48–50). For taxonomic identification, the 16S rRNA gene of selected colonies was sequenced and compared to the NCBI database. Notably, while the orange colonies were identified as belonging to the *Desulfuromonas* genus, we were not able to obtain them in an entirely pure culture, despite several rounds of isolation on solid media; we thus considered them as an enriched *Desulfuromonas* consortium, here referred to as EDC. For each MFC, several EDC colonies collected from the same petri dish were applied directly to the anode surface (i.e., bioaugmentation). To examine the microbial composition of the original consortium, EDC was sequenced via 16S rRNA amplicon sequencing and shotgun metagenomics. Both methods showed that Desulfuromonas acetexigens was the species most closely related to the *Desulfuromonas* enriched in EDC (this was verified by mapping shotgun metagenome reads to the *recA* gene that served as a taxonomic marker), as detailed in Fig. 2 and in Text S1.

10.1128/mBio.03629-20.9 TEXT S1 In-depth description of chemical measurements, DNA extraction, and microbial community data analysis. Download Text S1, DOCX file, 0.03 MB. Copyright © 2021 Yanuka-Golub et al. 2021 Yanuka-Golub et al. https://creativecommons.org/licenses/by/4.0/ This content is distributed under the terms of the Creative Commons Attribution 4.0 International license.

### DNA extraction and microbial community analysis.

Anodic biofilms and planktonic cells from the MFC systems were sampled at different time points for community and phylogenetic analyses, as previously described (19). For treatment groups EDC+WW, EDC+FWW, and WW, planktonic cells were sampled at three time points (day 1, day 3, and time at which steady-state current density production was achieved [SS]; *N *= 2). Day zero was considered either filtered or unfiltered inoculum source. For 16S rRNA amplicon, 72 samples were amplified using the 341F-806R primer set, sequenced, and analyzed (Text S1). An OTU table was used as input for phyloseq (51) and DESeq2 (52) R packages. Bacterial diversity within samples (alpha diversity) was estimated using Richness and three diversity indices, Simpson, Shannon, and Pielou’s evenness. To investigate the enrichment of specific populations in the planktonic or in the anode’s microbial community, we performed the DESeq function in the DESeq2 R package to contrast the anodic and planktonic microbial communities of WW and EDC+WW. Metagenome assembly, gene prediction, and functional profiles were completed for six DNA samples (6 of the same 72 genomic DNA samples that were sequenced for the 16S amplicon). Additionally, the putative genome of *D. acetexigens* was assembled from four samples, and the average nucleotide identity (ANI) was calculated. The methods used for DNA extraction, 16S rRNA gene and shotgun metagenome sequencing, library construction, and metagenome assembly analysis are fully described in Text S1.

### Electrochemical measurements.

Voltage was measured across a fixed external resistor (R ext = 1,000 Ω). The current (I) was calculated according to Ohm’s law, I = E/R, which was normalized to anode surface area (current density, A m^−2^). For polarization curves, voltage was plotted as a function of current density (A m^−2^). A resistor box was used to set variable external loads while recording the voltage for each external resister applied, and the current was calculated using Ohm’s law. The voltage value was recorded at fixed time gaps (10 to 15 min) after steady-state conditions had been established under the specific external resistance employed.

### Chemical measurement.

Water samples were filtered prior to the chemical analysis, unless stated otherwise (Amicon Ultra 0.5 ml 10 K; Mercury Ltd., Israel). The methods used for all chemical analyses of water samples can be found in Text S1.

### Statistical analyses.

Statistical analyses (*t* test and analysis of variance [ANOVA]) were performed using GraphPad Prism version 5.00 for Windows (GraphPad Software, San Diego, CA, USA).

### Data availability.

Metagenomic sequence data generated in this study have been deposited to NCBI SRA under BioProject ID PRJNA594175. 16S amplicon sequencing data have been submitted to the European Nucleotide Archive under the study with accession number PRJEB23191.
