# Supplementary material for: Anode Surface Bioaugmentation Enhances Deterministic Biofilm Assembly in Microbial Fuel Cells
Source: mBio. 2021 Mar 2;12(2):e03629-20. doi: 10.1128/mBio.03629-20 (PMC8092319; doi:10.1128/mBio.03629-20)
Supplement: FIG S1 [file mBio.03629-20-sf001.pdf]

A

B

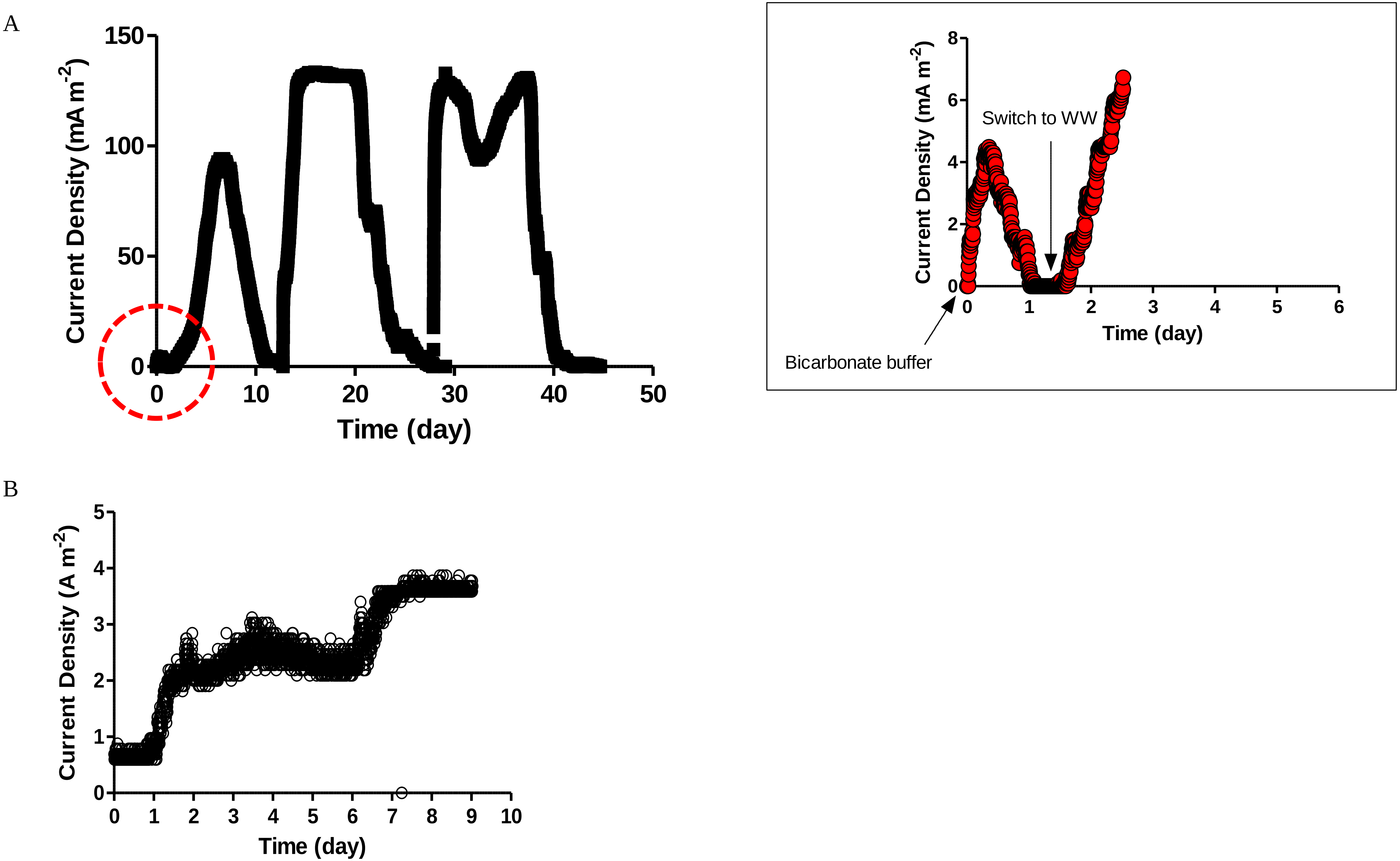

Figure S1A: Electrochemical behavior of the MFCs indicating the time at which the bulk liquid was changed.

Figure S1B: Electrochemical behavior of control MFC reactor inoculated with filtered wastewater and a clean anode.
