# Supplementary material for: Anode Surface Bioaugmentation Enhances Deterministic Biofilm Assembly in Microbial Fuel Cells
Source: mBio. 2021 Mar 2;12(2):e03629-20. doi: 10.1128/mBio.03629-20 (PMC8092319; doi:10.1128/mBio.03629-20)
Supplement: FIG S2 [file mBio.03629-20-sf002.pdf]

Figure S2:

A) BOD and COD analysis results for the sterile bicarbonate buffer (BCM), wastewater and filtered wastewater (10% v/v) used for inoculation of MFCs.

B) Nitrogen and phosphate concentrations measured for the sterile bicarbonate buffer (BCM), filtered wastewater (10% v/v) and wastewater (10% v/v).

C) Annual wastewater concentrations of BOD, COD and TSS reported by the wastewater treatment plant.

D) Taxonomic composition (phylum level) of wastewater samples taken for experiments 1-3.

E) Taxonomic composition (Proteobacteria-class level) of wastewater samples taken for experiments 1-3.

F) Alpha and Beta Diversity for the wastewater samples taken for inoculation of experiments 1-3.

A

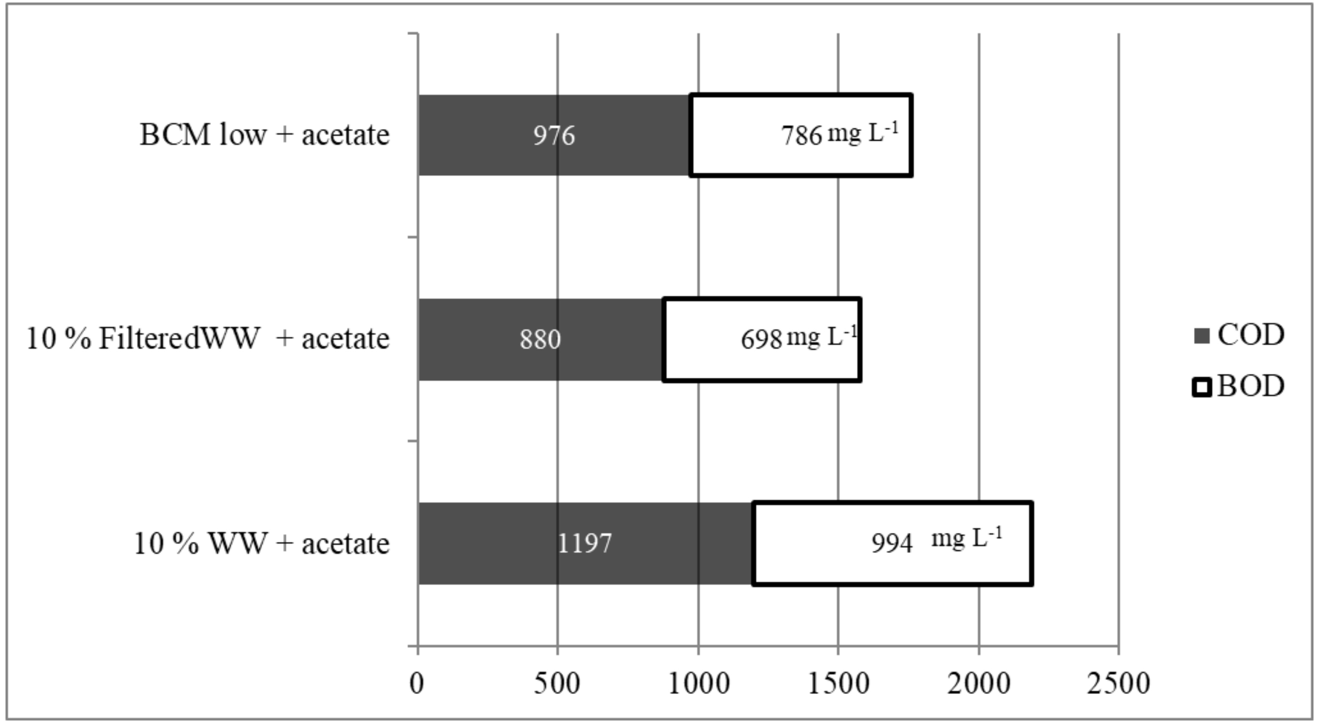

B

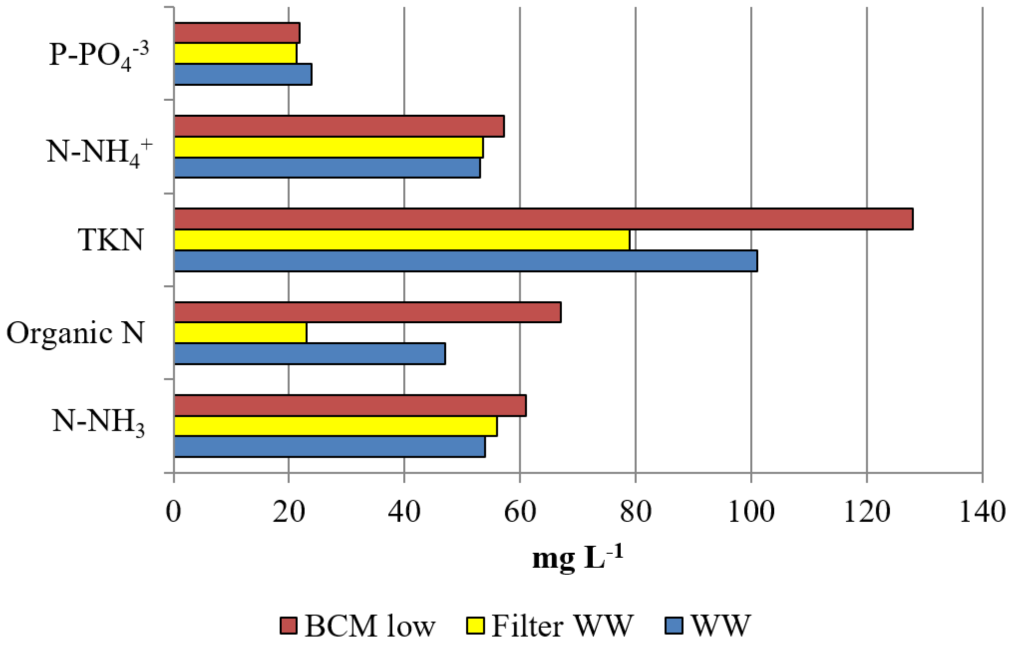

C

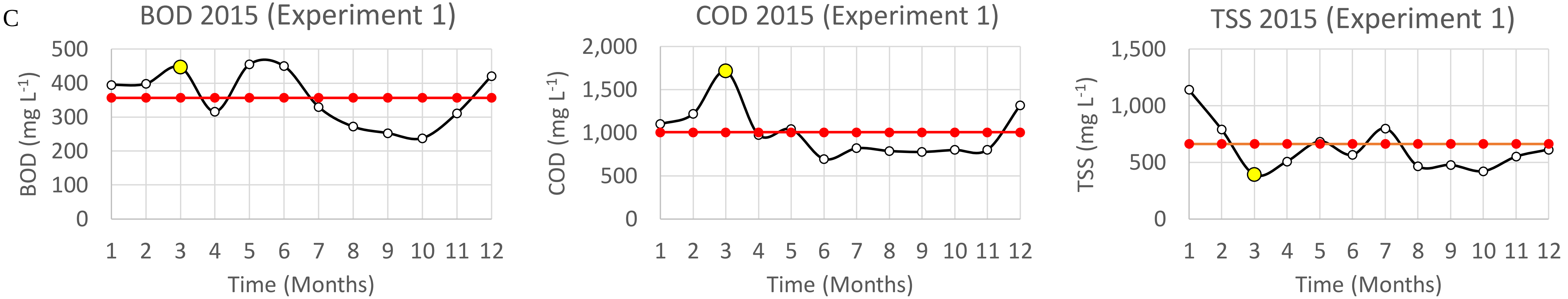

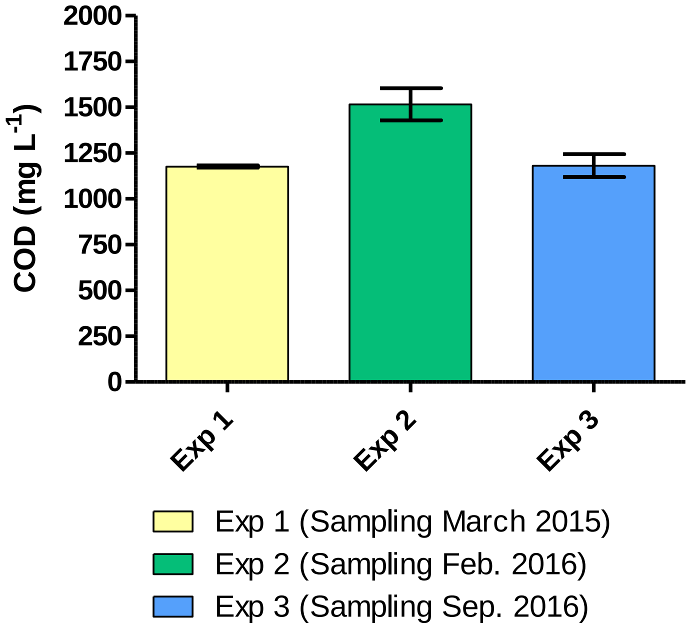

Upper panel: Annual wastewater concentrations of BOD, COD and TSS reported by the wastewater treatment plant (in Hebrew).

Bottom panel: Initial COD concentrations in the inoculated MFCs (diluted wastewater 10% V/V supplemented with acetate (20 mM), experiments 1-3.

D

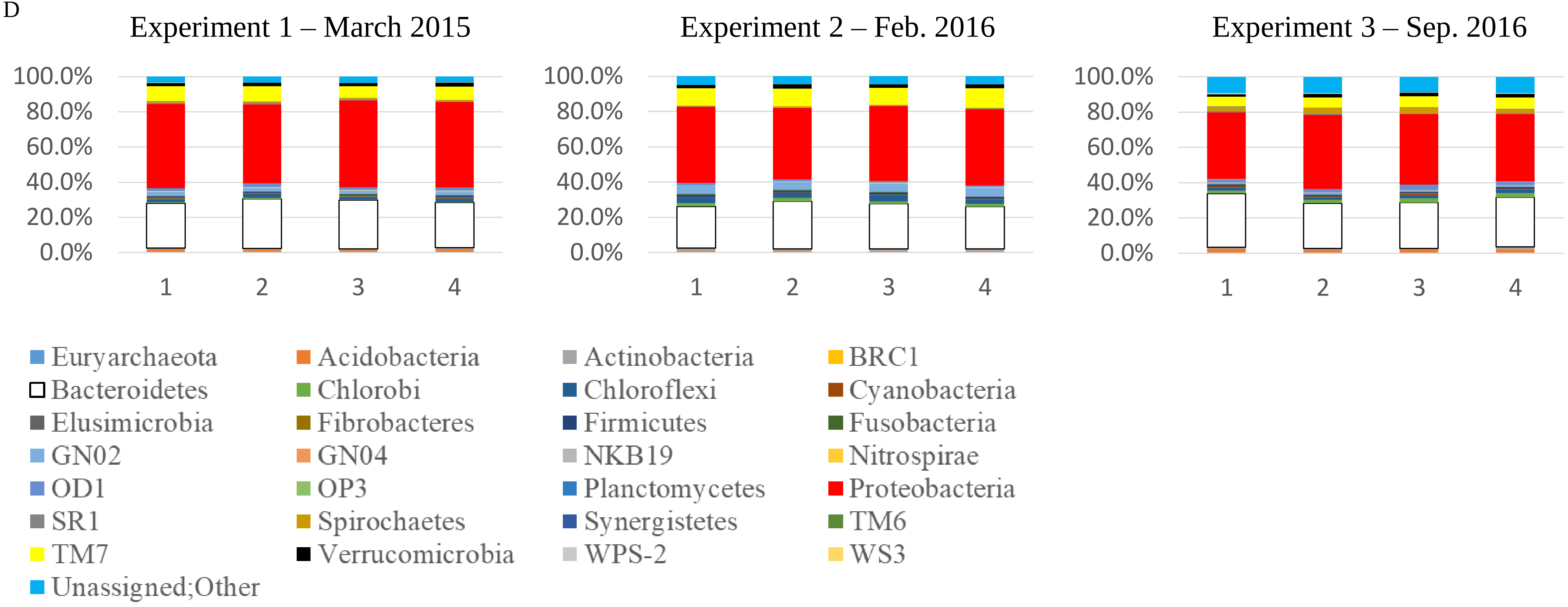
Experiment 1 – March 2015
Experiment 2 – Feb. 2016
Experiment 3 – Sep. 2016
100.0%
80.0%
60.0%
40.0%
20.0%
0.0%
1
2
3
4
Euryarchaeota
Acidobacteria
Actinobacteria
BRC1
Bacteroidetes
Chlorobi
Chloroflexi
Cyanobacteria
Elusimicrobia
Fibrobacteres
Firmicutes
Fusobacteria
GN02
GN04
NKB19
Nitrospirae
OD1
OP3
Planctomycetes
Proteobacteria
SR1
Spirochaetes
Synergistetes
TM6
TM7
Verrucomicrobia
WPS-2
WS3
Unassigned;Other

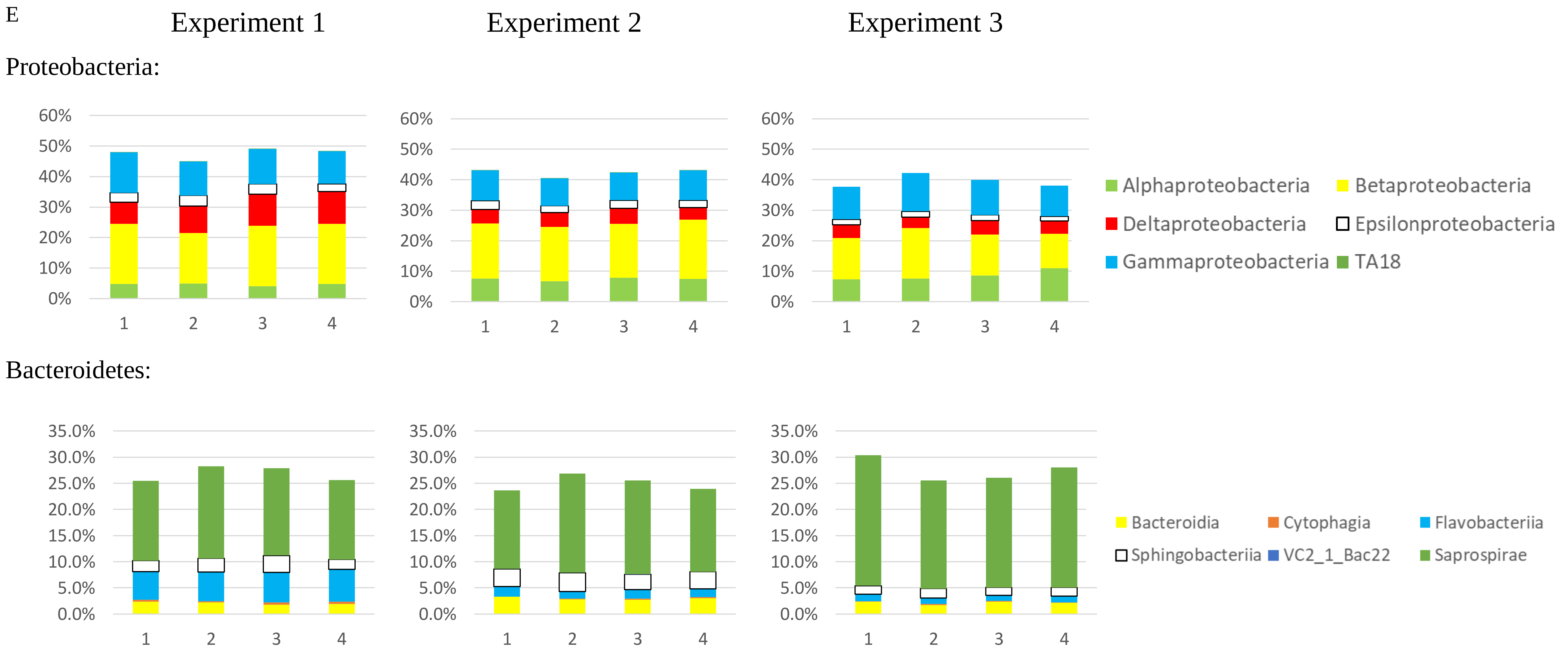
E
Experiment 1
Experiment 2
Experiment 3
Proteobacteria:
60%
50%
40%
30%
20%
10%
0%
1
2
3
4
Alphaproteobacteria
Betaproteobacteria
Deltaproteobacteria
Epsilonproteobacteria
Gammaproteobacteria
TA18
Bacteroidetes:
35.0%
30.0%
25.0%
20.0%
15.0%
10.0%
5.0%
0.0%
Bacteroidia
Cytophagia
Flavobacteriia
Sphingobacteriia
VC2_1_Bac22
Saprospirae

F

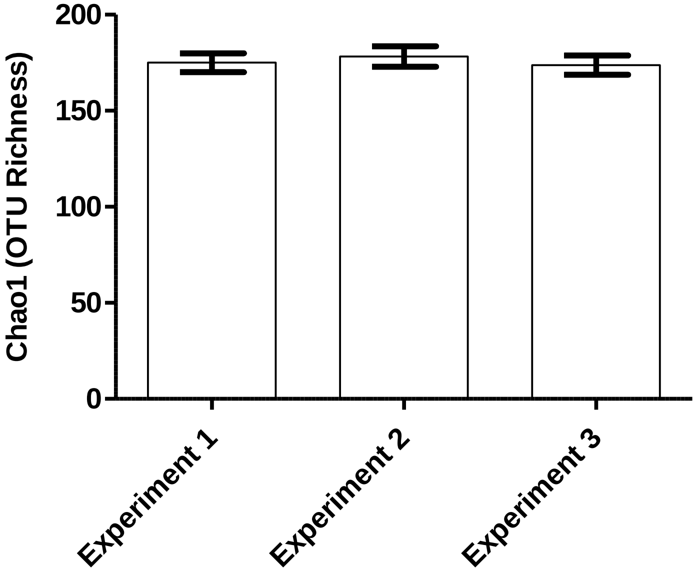

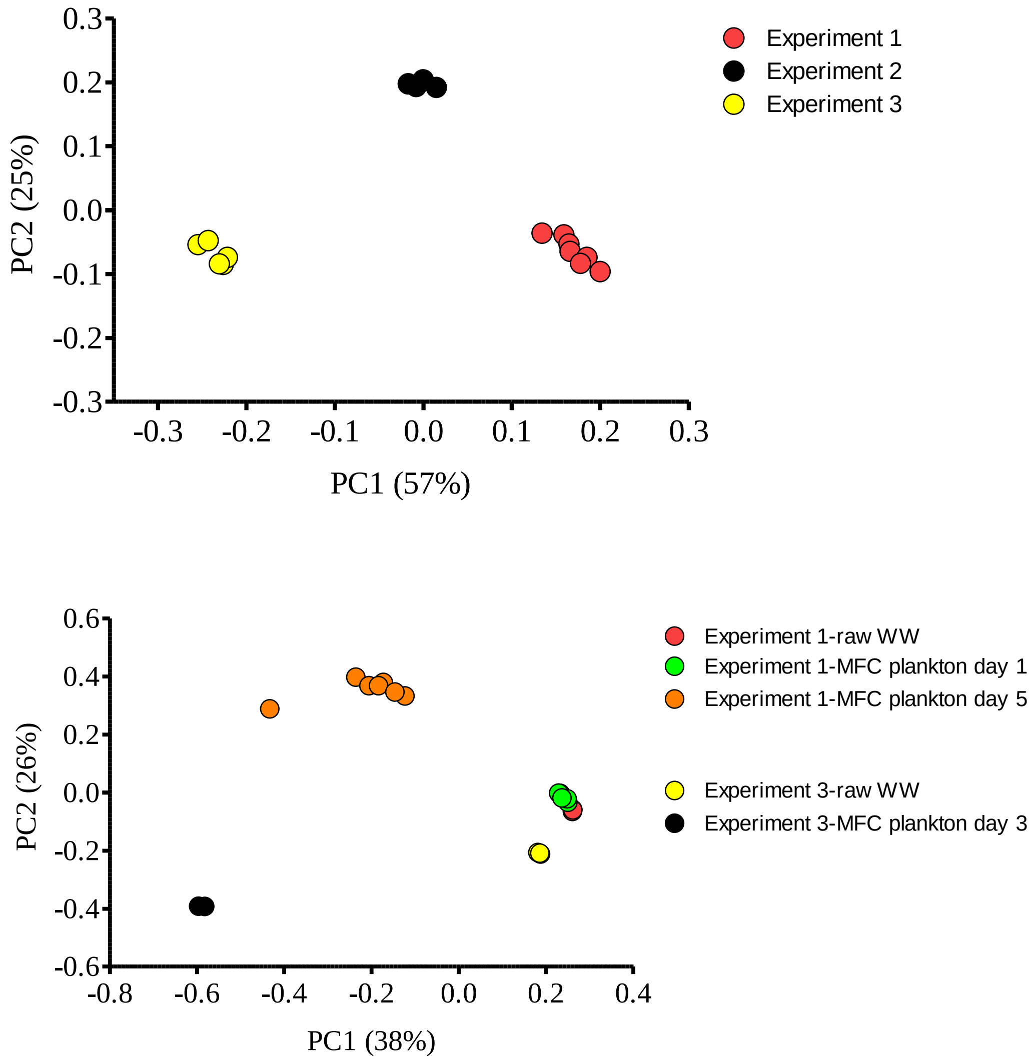

(Left): Chao species richness index of all replicate wastewater samples used for inoculating all MFCs in experiments 1-3. The diversity indiex was calculated with the PAST statistics software program.

(Right): Principal coordinates analysis (PCoA) based on the Bray-Curtis distances between microbial communities of the wastewater samples used for the three sets of experiments and temporal planktonic samples, as indicated by the different colors. PC, principle coordinate.
