# Supplementary material for: Anode Surface Bioaugmentation Enhances Deterministic Biofilm Assembly in Microbial Fuel Cells
Source: mBio. 2021 Mar 2;12(2):e03629-20. doi: 10.1128/mBio.03629-20 (PMC8092319; doi:10.1128/mBio.03629-20)
Supplement: FIG S3 [file mBio.03629-20-sf003.pdf]

A

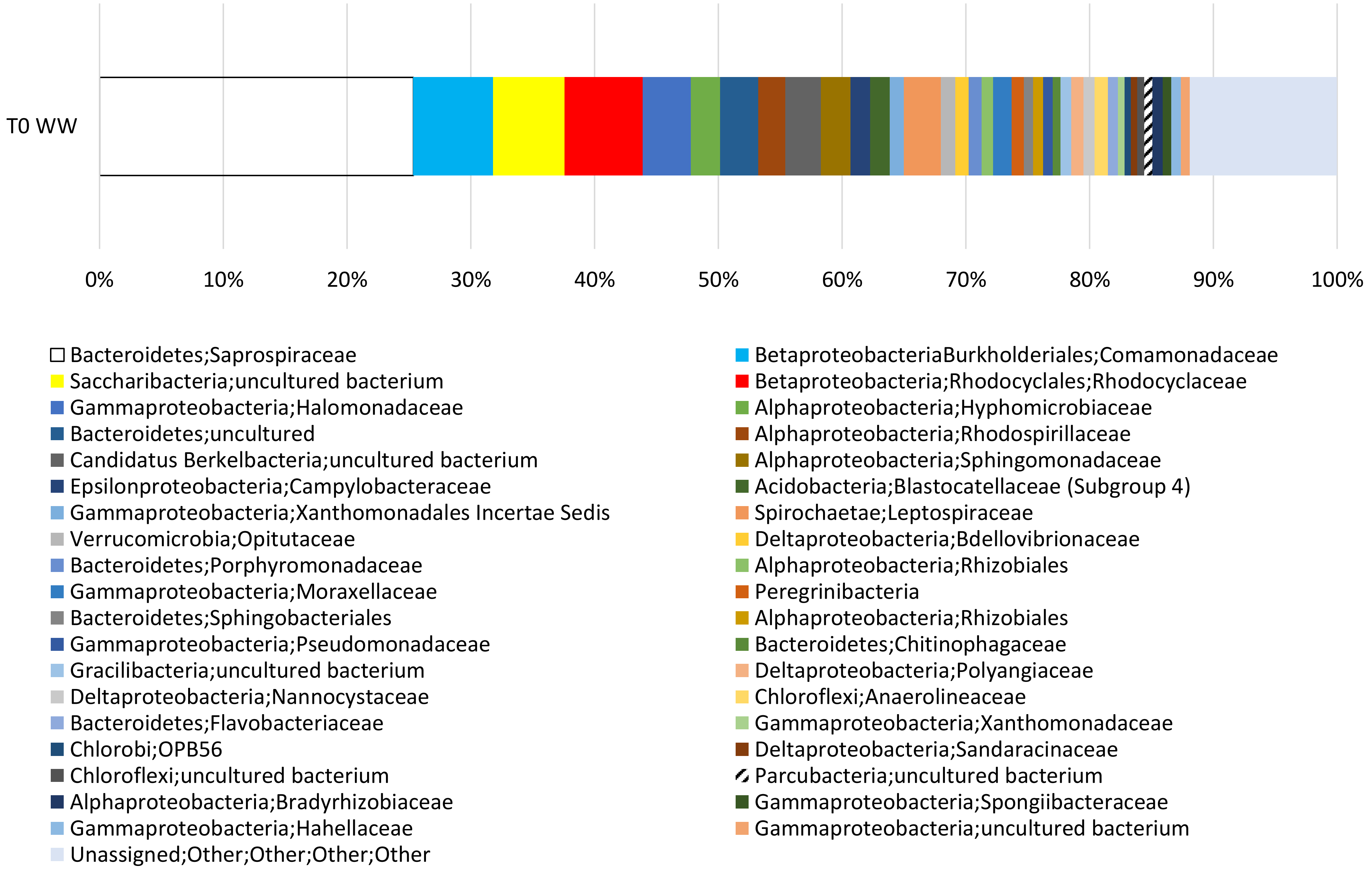

B

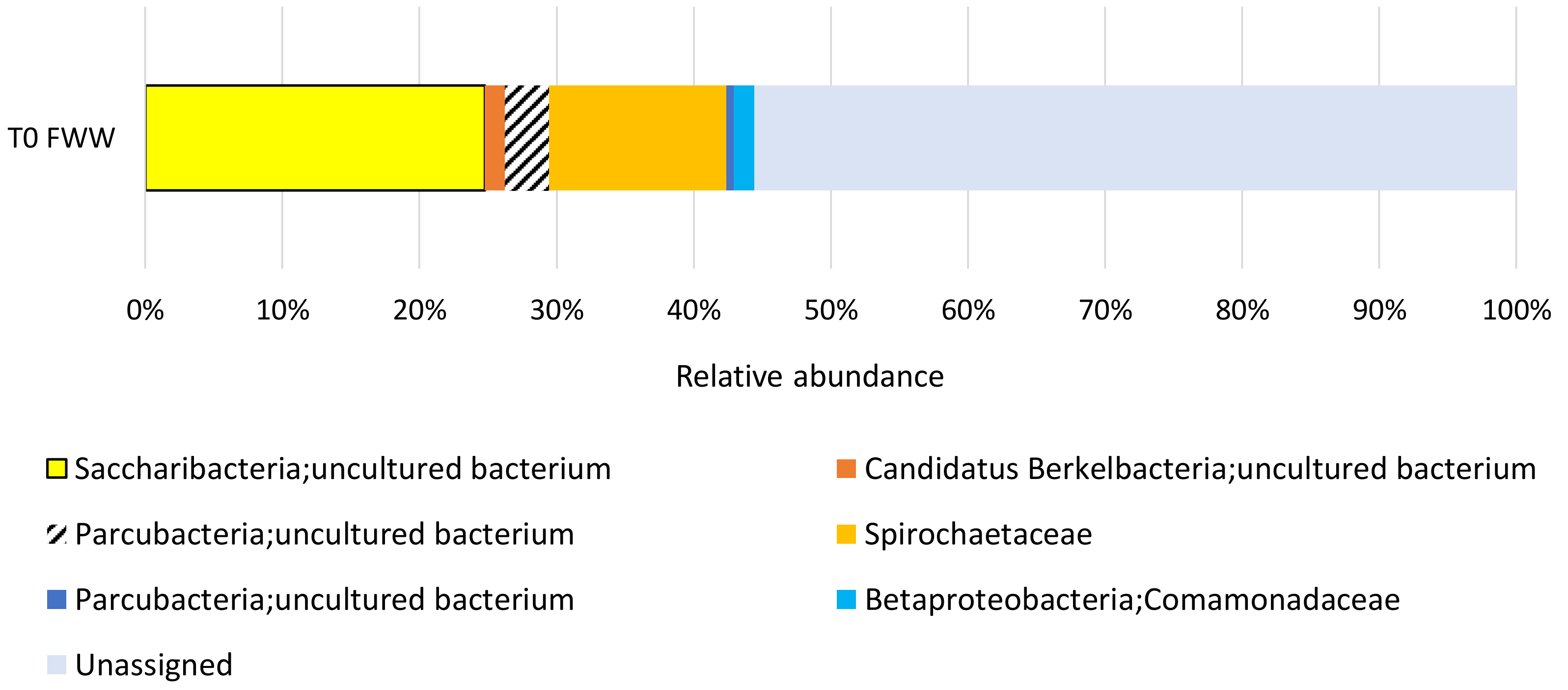

Figure S3: A) Distribution of taxa in the **unfiltered** wastewater solution used for inoculating the MFCs. B) Distribution of taxa in the **filtered** wastewater solution used for inoculating the MFCs. Bacterial relative abundances is shown for both plots at the Family level via 16S rRNA gene sequencing. Both solutions were supplemented with acetate and diluted in BCM.
