# Supplementary material for: Anode Surface Bioaugmentation Enhances Deterministic Biofilm Assembly in Microbial Fuel Cells
Source: mBio. 2021 Mar 2;12(2):e03629-20. doi: 10.1128/mBio.03629-20 (PMC8092319; doi:10.1128/mBio.03629-20)
Supplement: FIG S4 [file mBio.03629-20-sf004.pdf]

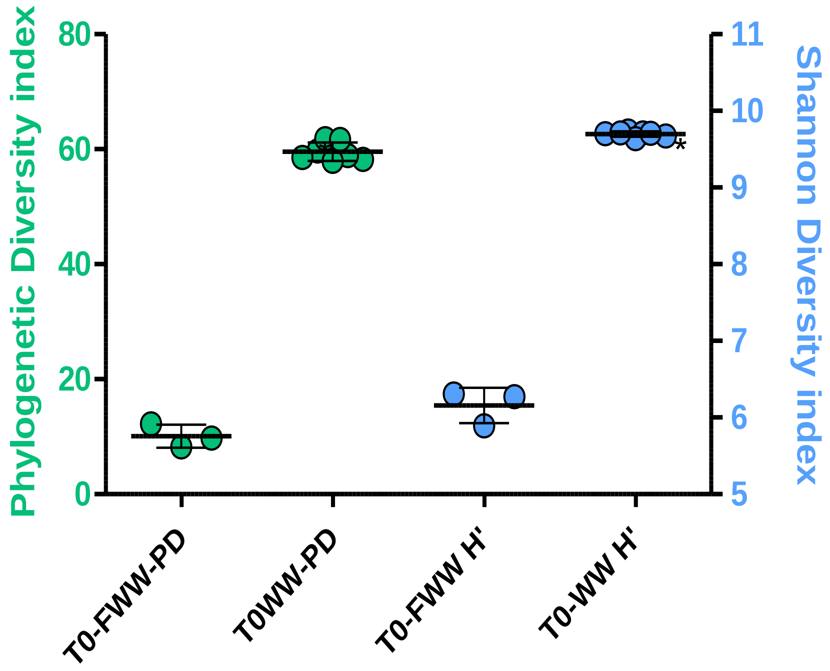

Figure S4: Shannon and Phylogenetic Diversity indices of the filtered and unfiltered wastewater solutions used for MFCs inoculation.
