# Supplementary material for: Anode Surface Bioaugmentation Enhances Deterministic Biofilm Assembly in Microbial Fuel Cells
Source: mBio. 2021 Mar 2;12(2):e03629-20. doi: 10.1128/mBio.03629-20 (PMC8092319; doi:10.1128/mBio.03629-20)
Supplement: FIG S5 [file mBio.03629-20-sf005.pdf]

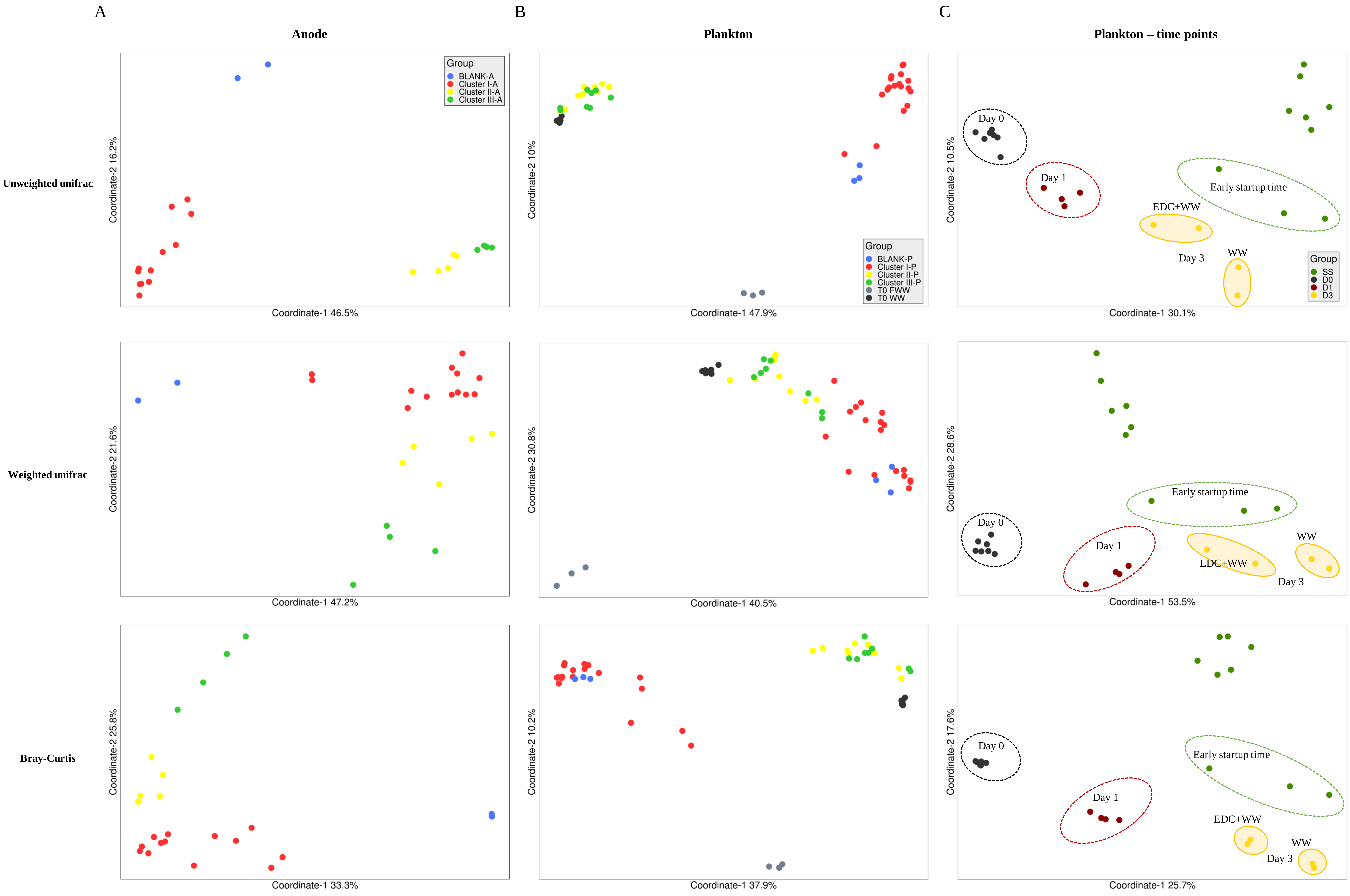

Figure S5:Principal Coordinate Analysis (OTU level) based on Unifrac (weighted or unweighted), or Bray-Curis distances (each PCoA uses one measure) of the A) anode-biofilm communities: BLANK: control of filtered wastewater with a clean anode, Cluster I: EDC, EDC+B, EDC+FWW, EDC+B—WW, Cluster II: EDC+WW, Cluster III: control – MFC supplemented with wastewater (WW) and clean anode B) plankton communities and C) plankton communities of only clusters II and III at specific time points: day 0 at which wastewater was supplemented with the bioaugmented anode, D1: day 1 after inoculation, D3: day 3, SS: Steady-State – the time at which stable maximal current density was achieved, which varied for each MFC, depending on when the last electrochemical cycle terminated.
