# Supplementary material for: Anode Surface Bioaugmentation Enhances Deterministic Biofilm Assembly in Microbial Fuel Cells
Source: mBio. 2021 Mar 2;12(2):e03629-20. doi: 10.1128/mBio.03629-20 (PMC8092319; doi:10.1128/mBio.03629-20)
Supplement: FIG S7 [file mBio.03629-20-sf007.pdf]

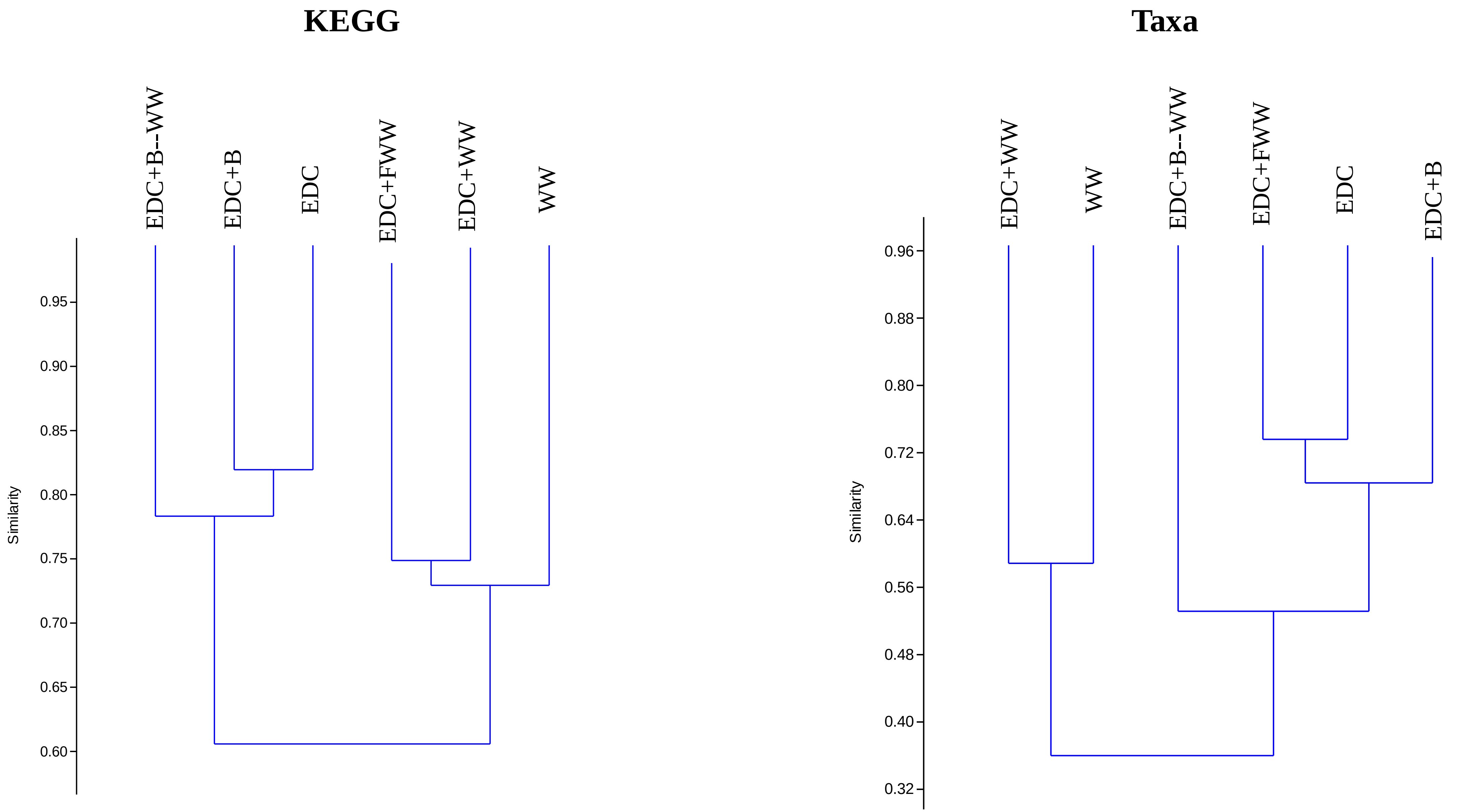

Figure S7: Cluster analysis of the six samples sequenced via shotgun metagenomics for the KEGG ortholog functions and taxonomic classifications. Clustering was computed using the Bray-Curtis dissimilarity index.
