# Supplementary figures and images for: Anode Surface Bioaugmentation Enhances Deterministic Biofilm Assembly in Microbial Fuel Cells
Source: mBio. 2021 Mar 2;12(2):e03629-20. doi: 10.1128/mBio.03629-20 (PMC8092319; doi:10.1128/mBio.03629-20)

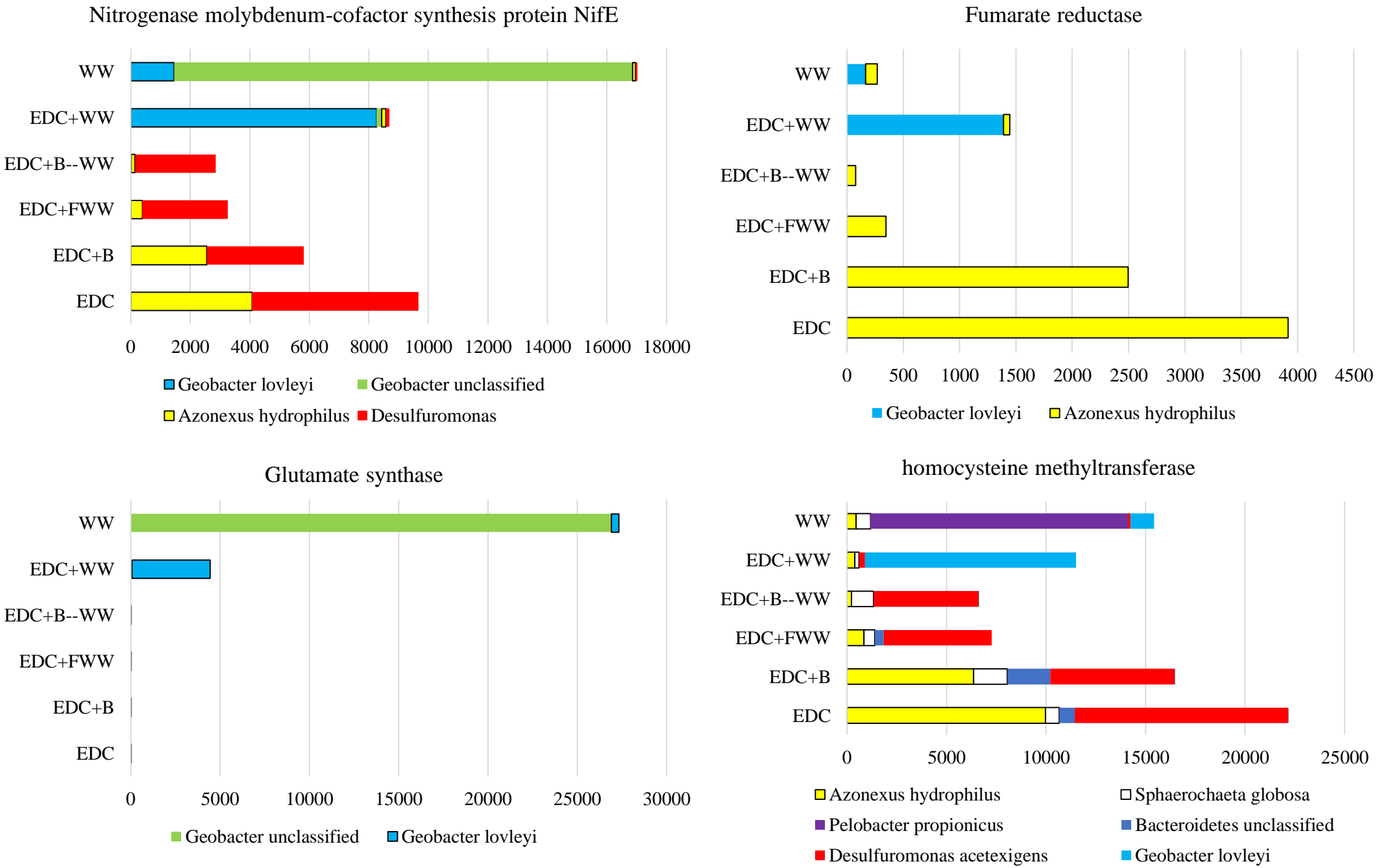

**Figure S8:** Taxonomic affiliation of four KEGG ortholog functions.

Supplement: FIG S8 [file mBio.03629-20-sf008.pdf]
